# Therapeutic Targets for the Treatment of Cardiac Fibrosis and Cancer: Focusing on TGF-β Signaling

**DOI:** 10.3389/fcvm.2020.00034

**Published:** 2020-03-10

**Authors:** Warisara Parichatikanond, Theerut Luangmonkong, Supachoke Mangmool, Hitoshi Kurose

**Affiliations:** ^1^Department of Pharmacology, Faculty of Pharmacy, Mahidol University, Bangkok, Thailand; ^2^Department of Pharmacology, Faculty of Science, Mahidol University, Bangkok, Thailand; ^3^Department of Pharmacology and Toxicology, Graduate School of Pharmaceutical Sciences, Kyushu University, Fukuoka, Japan

**Keywords:** anticancer, antifibrotic, cancer, cardiac fibrosis, inhibitors of TGF-β signaling, transforming growth factor-β (TGF-β)

## Abstract

Transforming growth factor-β (TGF-β) is a common mediator of cancer progression and fibrosis. Fibrosis can be a significant pathology in multiple organs, including the heart. In this review, we explain how inhibitors of TGF-β signaling can work as antifibrotic therapy. After cardiac injury, profibrotic mediators such as TGF-β, angiotensin II, and endothelin-1 simultaneously activate cardiac fibroblasts, resulting in fibroblast proliferation and migration, deposition of extracellular matrix proteins, and myofibroblast differentiation, which ultimately lead to the development of cardiac fibrosis. The consequences of fibrosis include a wide range of cardiac disorders, including contractile dysfunction, distortion of the cardiac structure, cardiac remodeling, and heart failure. Among various molecular contributors, TGF-β and its signaling pathways which play a major role in carcinogenesis are considered master fibrotic mediators. In fact, recently the inhibition of TGF-β signaling pathways using small molecule inhibitors, antibodies, and gene deletion has shown that the progression of several cancer types was suppressed. Therefore, inhibitors of TGF-β signaling are promising targets for the treatment of tissue fibrosis and cancers. In this review, we discuss the molecular mechanisms of TGF-β in the pathogenesis of cardiac fibrosis and cancer. We will review recent *in vitro* and *in vivo* evidence regarding antifibrotic and anticancer actions of TGF-β inhibitors. In addition, we also present available clinical data on therapy based on inhibiting TGF-β signaling for the treatment of cancers and cardiac fibrosis.

## Introduction

**Figure 1:**
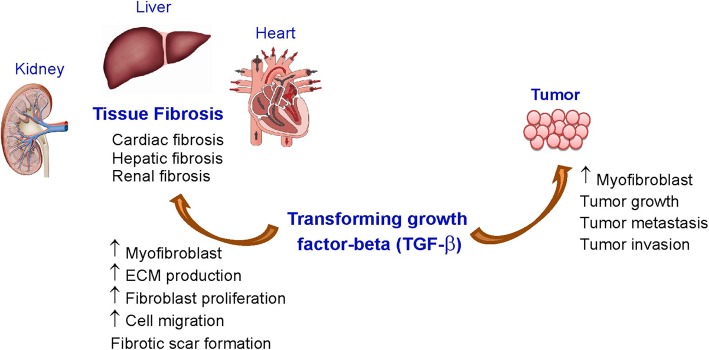
Effects of TGF-β on tissue fibrosis and cancer. ECM, extracellular matrix; TGF-β, transforming growth factor-beta-β.

Transforming growth factor-β (TGF-β) is a crucial member of the TGF-β superfamily and its sophisticated signaling pathways have pleiotropic effects that regulate several systems throughout the body such as cell growth, cell differentiation, apoptosis, motility and invasion, tissue remodeling, angiogenesis, and the immune response (1–6). TGF-β signaling dysfunctions are frequently found in tumors and these dysfunctions play critical roles in tumor progression (e.g., development and metastasis) (7–9). In addition, TGF-β is a major profibrotic mediator that plays an important role in the development of fibrosis (10). Due to the significant implication of TGF-β signaling in cancer as well as in fibrosis (Figure 1), drug research into treatments for cancer and fibrosis has aimed to develop various approaches to inhibit TGF-β signaling. Thus, the number of lead compounds used either in animal models or in clinical studies related to cancer and fibrosis is currently growing. Targeting TGF-β signaling pathways could be a novel therapeutic strategy to treat a variety of fibrotic disorders and cancers.

The synthesis and secretion of TGF-β, including its activity, is markedly increased in experimental models of fibrosis and in patients with tissue fibrosis (e.g., liver, lung, kidney, and heart). Fibrosis is an important pathophysiological phenomenon in many tissues. It is characterized by fibroblast activation and accumulation, an imbalance of extracellular matrix (ECM) production and degradation, and myofibroblast differentiation, which results in the accumulation of fibrotic scar and tissue stiffness, leading to distortions of organ architecture and function [Reviewed in (11, 12)].

Among fibrotic conditions in various organs, cardiac fibrosis is a major pathologic disorder associated with a great number of cardiovascular diseases resulting from an excessive ECM protein deposition in the heart [Reviewed in (11, 12)]. The etiologies of cardiac fibrosis and myocardial stiffness are multifactorially developed in response to multiple risk factors (13, 14) include myocardial infarction (MI), hypertension (15), diabetes (16, 17), aging (16), and excessive alcohol consumptions (18, 19) leading to the excessive deposition of ECM. After cardiac injury, alterations in ECM homeostasis, the upregulation and release of growth factors and cytokines, and differentiation of fibroblasts into myofibroblasts dynamically modulate cardiac fibroblast characteristics and functions, leading to myocardial fibrosis. Myocardial fibrosis is associated with fibrotic scar formation, myocardial stiffness, and the progression of heart failure (HF) (20–23). Treatment of HF and cardiac fibrosis still has limited efficacy and currently there is no drug approved for the treatment of cardiac fibrosis. The main reason is that the underlying mechanism of fibrosis is still unclear. However, cardiovascular diseases remain the leading global cause of death (22, 23) and understanding the pathogenesis of fibrotic myocardial remodeling is crucial to identifying innovative treatment strategies for patients with cardiac fibrosis.

In the heart, activation of cardiac fibroblasts mainly by TGF-β leads to alterations in cardiac ECM and cardiac remodeling that play a major role in the development and progression of heart diseases (10, 22). A significant number of preclinical and clinical studies have reported that inhibition of TGF-β signaling pathways by various strategies exhibited potential effectiveness for the treatment of cardiac fibrosis. Cancers and fibrotic diseases share the most common pathologies associated with the activity of TGF-β (1, 2). Here, we review the molecular mechanisms and signaling pathways of TGF-β and their effect on cancer and cardiac fibrosis, and we also summarize the role of inhibition of TGF-β for anticancer and antifibrotic therapies.

### Introduction of Cancer

Cancer is defined as a collection of diseases relating to atypical cell growth. In physiological process, new cells can grow, divide, and replace senescent or damaged cells. However, this systemically process fails when cancer develops as aged or injured cells remain survive, together with a proliferation of unneeded new cells. These unnecessary cells can divide, spread, and invade nearby tissues without stopping. Also, the harm cells can possibly travel through the blood or lymph system to invade remote tissues. This atypical cell growth and spreading is known as carcinogenesis (24). Widespread and recognized theory of carcinogenesis is the DNA mutations that disrupt the normal balance between proliferation and cell death. Variants of inherited genes and environmental factors might play a pivotal role in DNA mutations. In addition, viruses containing oncogenes are recently known as a trigger of cancer cell growth (24).

#### Therapeutic Targets for Treatment of Cancers

Treatment of cancers can be achieved using several strategies such as surgery, radiation, and especially drugs. Chemotherapy is a conventional treatment by using toxic drugs to kill cancer cells. Beyond fast-growing cancer cells, traditional anticancer drugs using for chemotherapy damage healthy cells that rapidly grow and divide, leading to multiple adverse effects (25). Newer drugs for the treatment of cancers were subsequently developed for a preferable safety issue and prevailing therapeutic efficacy (25). Hormonal therapy is another strategy to cease the growth of cancer which required certain hormones. Due to the blockade, undesired effects of anti-hormone drugs can be seen depending on types of interfered hormone (26, 27). Targeted therapy is a type of cancer treatment using drugs targeting particular molecules required for the pathogenesis of individual cancer. Nevertheless, treated cancer cells can gradually resist to targeted therapy, and conventional chemotherapy might be needed to be co-administered in the regimen for a better outcome (28). Immunotherapy is a novel treatment method by enhancing immune system for eradicating cancer cells. Despite solely activated self-immune cells, overactive immunity against cancer also influences healthy cells and tissues resulting in various adverse effects (29). Described anticancer drug classes and representative drugs among each class are demonstrated in Table 1. However, in-depth review regarding mechanism of drug action, clinical effectiveness, and safety profile of these anticancer drugs are beyond our scope. Furthermore, it should be noted that although anticancer drugs appears to be diverse and abundant, we still need distinct agents to deal with innumerable types of advanced cancers in clinical practice, especially multi-drug resistant cancers (30). Therefore, in this review, we focus on the role of TGF-β and its signaling on the treatment of cancer.

**Table 1:** Available anticancer drug classes and representative drugs among each class.

**Classes**	**Example sub-classes**	**Representative drugs**
Chemotherapy (25)	Alkylating agents	Cyclophosphamide, cisplatin
	Topoisomerase inhibitors	Irinotecan, etoposide, doxorubicin
	Mitotic inhibitors	Vincristine, paclitaxel
	Anti-metabolites	Methotrexate, cytarabine, hydroxyurea
	Others	Bleomycin, L-asparaginase
Hormonal therapy (26, 27)	GnRH analogs	Buserelin, degarelix
	Anti-androgens	Cyproterone, flutamide
	Aromatase inhibitors	Aminoglutethimide, anastrozole
	SERMs	Tamoxifen
Targeted therapy (28)	Receptor tyrosine kinase inhibitors	Erlotinib, gefitinib, lapatinib
	Intracellular tyrosine kinase inhibitors	Imatinib, nilotinib, everolimus
	Phenotype-directed inhibitors	Rituximab, alemtuzumab
	Ligand-receptor binding inhibitors	Bevacizumab, cetuximab, trastuzumab
	Proteasome inhibitors	Bortezomib
Immunotherapy (29)	PRR agonists	Imiquimod, mifamurtide
	Checkpoint inhibitors	Ipilimumab, nivolumab
	Cytokines	IFN-α, IFN-β
	Cell-based immunotherapies	Sipuleucel-T

*GnRH, gonadotropin releasing hormone; IFN, interferon; PRR, pattern recognition receptor; SERMs, selective estrogen receptor modulators*.

### Introduction of Cardiac Fibrosis

Cardiac fibrosis is a pathological remodeling process following cardiac injury, MI, and other heart diseases. Cardiac fibrosis disrupts the communication and function of myocytes and non-myocyte cells in the heart, leading to contractile dysfunction and arrhythmia. Fibrosis also accelerates the remodeling processes that exhibit detrimental effects on the heart (23, 31).

The imbalance between production and degradation of interstitial ECM proteins leads to progressively increased cardiac stiffness and diastolic dysfunction (23). Lines of existed evidence demonstrates that the pathogenesis of diastolic dysfunction caused by cardiac fibrosis (32, 33). In the fibrotic heart, collagens mainly from activated myofibroblasts undergoes cross-linking process contributing to the progression of diastolic dysfunction and the restricted cardiac chamber compliance (34, 35). In addition, ECM overproduction and deposition between the layers of cardiac myocytes results in the disruption of myocardial electrophysiological functions, which leads to contractile dysfunction and an increased risk of cardiac arrhythmia (36, 37). In fact, TGF-β induced cardiac fibrosis is seriously involved in the pathogenesis of arrhythmia by disturbing electrical signal conduction, leading to the generation of re-entry circuits (10).

#### Myofibroblasts

**Figure 2:**
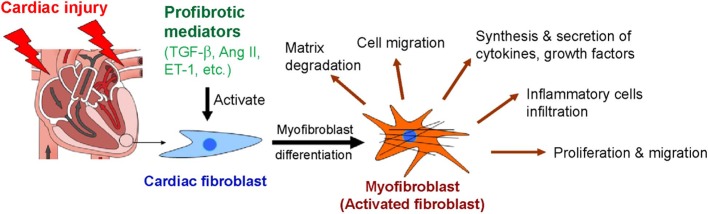
Myofibroblast differentiation and functions of myofibroblasts after cardiac injury. Ang II, angiotensin II; ET-1, endothelin-1; TGF-β, transforming growth factor-β.

In the heart, cardiac fibroblasts can be transdifferentiated into myofibroblasts with contractile, migratory, and secretory properties (Figure 2). Myofibroblast is a key regulator that accelerates the fibrotic response in many conditions associated with HF. Regardless of the etiology of cardiac fibrosis, myofibroblast transdifferentiation is a hallmark of the fibrotic response in the heart [Reviewed in (20, 23)].

Myofibroblasts are the activated form of fibroblasts. They overexpress α-smooth muscle actin (α-SMA) and contain contractile bundles of actin filaments resembling the myofibrils of smooth muscle cells and associated proteins organized into prominent stress fibers (38). The incorporation of α-SMA into contractile bundles is a major characteristic of differentiated myofibroblasts and significantly increases contractile function. Thus, α-SMA has been suggested to be the most significant marker of myofibroblasts (39). Although α-SMA is found in human myocardial scars, the other structural ECM proteins such as collagens, vimentin, and desmin are also present in fibrotic scars (40). Fibroblast differentiation into myofibroblast is controlled by a variety of growth factors and cytokines. Among them, TGF-β is a strong inducer that stimulates myofibroblast formation (Figure 2).

Fibroblasts are abundant in normal hearts and can differentiate into myofibroblasts via profibrotic mediators such as TGF-β (41, 42). This process suggests that the activation of resident fibroblasts represents a major source of myofibroblasts in hearts with fibrosis. In addition, proliferating myofibroblasts are commonly found in high numbers in the infracted area of the heart (41, 42).

Following cardiac fibroblast activation, inflammatory cells (e.g., macrophages, monocytes, and mast cells) infiltrate the site of remodeling myocardium and secrete various types of profibrotic mediators, including growth factors and cytokines [Reviewed in (43)]. These mediators have been found to promote myofibroblast formation, but the most significant and common inducer is TGF-β (44). TGF-β accelerates the differentiation of resident fibroblasts, epithelial cells, and endothelial cells into myofibroblasts (44). Thus, agents that inhibit myofibroblast differentiation might provide a tool to prevent the maladaptive myocardial remodeling that occurs in response to profibrotic stimuli and for fibrosis prevention.

#### Overproduction of ECM Proteins

Alterations in ECM homeostasis, especially in terms of ECM overproduction, lead to cardiac dysfunction. Several mediators, including angiotensin II (Ang II), and TGF-β, regulate ECM production by cardiac fibroblasts (45). In response to cardiac injury, myocardial fibrosis results from an imbalance of both ECM synthesis and degradation, leading to an accumulation of collagen type I and III in the heart (20, 23). Deposition of ECM proteins is significantly increased in the hearts of patients with cardiac diseases (46). In addition, the levels of cardiac fibrosis are associated with cardiac dysfunction (46). Moreover, ECM deposition and fibroblast activation contribute to the impairment of ventricular compliance and filling due to increased ventricular stiffness (20, 23). Furthermore, overproduction of ECM interrupts the electrophysiological functions in the heart, leading to arrhythmias (10).

#### Therapeutic Targets for Treatment of Cardiac Fibrosis

According to cardiac fibrosis is associated with cardiac remodeling and is involved in the pathogenesis of HF, the prevention and reversal of cardiac fibrosis is an important therapeutic target for the treatment of HF. Numerous signaling pathways, through a variety of profibrotic mediators (e.g., Ang II, endothelin-1 [ET-1], and TGF-β), have been implicated in the activation of cardiac fibroblasts and the development of cardiac fibrosis. Modulation of these signaling pathways using inhibitors is of great interest for the treatment and prevention of cardiac fibrosis. Below, we summarize the update and important roles of several agents that act against cardiac fibrosis (Table 2). Although, both angiotensin converting enzyme inhibitors (ACEIs) and angiotensin II receptor blockers (ARBs) have already demonstrated significant efficacy in reducing cardiac fibrosis in human and animal models of HF, neither ACEIs nor ARBs have been approved for the treatment of cardiac fibrosis. Further studies are required to establish the molecular mechanisms of ACEIs and ARBs not only for treatment but also for reversal of fibrotic remodeling in HF.

**Table 2:** Therapeutic targets/strategies for treatment of cardiac fibrosis.

**Targets/Strategies**	**Results**	**References**
Inhibitors of TGF-β and its signaling pathway	Anti-TGF-β neutralizing antibody prevents myocardial fibrosis in pressure-overloaded hearts	(47)
	Blockade of TGF-β-activated kinase 1 (TAK1) inhibits TGF-β-mediated extracellular matrix (ECM) overproduction in cardiac fibroblasts	(48)
	Inhibition of p38-MAPK suppresses TGF-β-induced myofibroblast activation and ECM production	(49)
TβRI (ALK5) inhibitors	ALK5 inhibition attenuates cardiac dysfunction and remodeling after myocardial infarction (MI)	(50)
	SM16 (ALK5 inhibitor) attenuates progression of cardiac fibrosis in left ventricular (LV) pressure overload	(51)
TβRII inhibitors	Dominant negative mutant of TβRII inhibits interstitial fibrosis in pressure-overload hearts	(52)
Smad inhibitors	Halofuginone (Smad3 inhibitor) attenuates radiation-induced fibrosis	(53)
Angiotensin converting enzyme inhibitors/angiotensin II receptor blockers (ACEIs/ARBs)	Losartan inhibits the progression of cardiac hypertrophy and fibrosis	(54)
	Lisinopril improves cardiac function and attenuates fibrosis in patients with hypertension and hypertrophy	(55)
	Losartan reduces angiotensin II (Ang II)-induced collagen synthesis and fibroblast activation	(56)
Endothelin receptor (ETR) antagonists	Bosentan improves cardiac function and reduces infarct size in a rat model of ischemia/reperfusion injury	(57)
	ET_A_R antagonists prevented cardiac fibrosis in hypertensive-induced rats	(58)
Adenosine receptor (AR) agonists	Stimulation of A_2B_R attenuates fibrosis and remodeling in a rat model of MI	(59)
	Stimulation of A_2B_R inhibits ET-1-induced fibroblast proliferation and α-SMA synthesis	(60)
	Stimulation of A_2B_R inhibits Ang II-induced collagen synthesis and myofibroblast differentiation	(61)
β-Adrenergic receptor (βAR) signaling	Blockade of βAR attenuates cardiac fibrosis in an animal model of heart failure (HF)	(62)
	Gene deletion of GRK2 enhances survival, improves contractility, and inhibits cardiac remodeling in a mouse model of post-MI	(63)
	Treatment with β-blockers (e.g., atenolol, metoprolol, and propranolol) blocked the effects of βAR-mediated fibroblast activation	(64)

## TGF-β Signal Transduction

TGF-β is a member of the TGF-β superfamily, which is comprised of TGF-β, bone morphogenetic proteins (BMPs), growth differentiation factors (GDFs), activin and inhibin (65). Members of this diversify superfamily are the pleiotropic multifunctional polypeptides that play a role in a wide range of physiological cellular activities such as growth, proliferation, differentiation, and apoptosis (65). Among these polypeptides, TGF-β has been proven to be one of the major factors driving the fibrotic response in most organs (2). In mammals, there are 3 isoforms of TGF-β: TGF-β1, TGF-β2, and TGF-β3. These highly homologous polypeptides, encoded by various genes, are synthesized, processed and regulated in a similar fashion. However, these 3 isoforms are secreted by various types of cells and signals through the same receptors, but they exhibit distinct patterns of distribution in different tissues (3, 66). Even though any isoform can be found in fibrotic tissues, the progression of organ fibrosis, in particular cardiac fibrosis, is predominantly attributed to TGF-β1 (67). To date, information on isoform-specific activities of various isoforms of TGF-β in a specific pathology is lacking and needs further investigation. Next, the signaling of TGF-β, excluding conclusions regarding specific isoforms, is discussed in detail.

**Figure 3:**
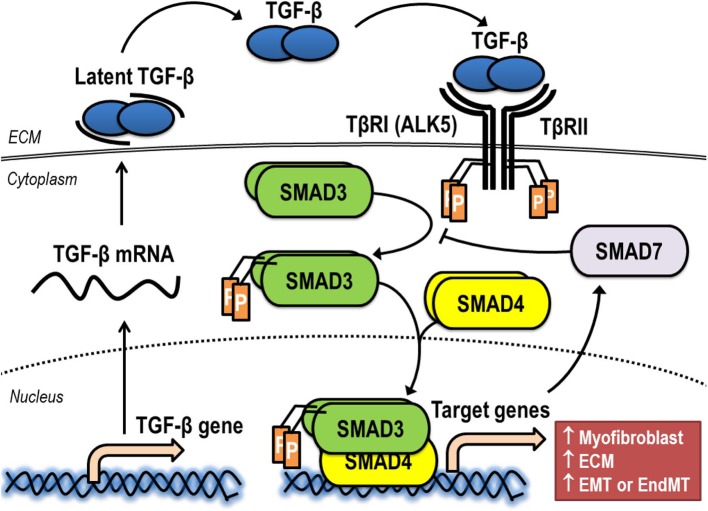
Synthesis, release, and activation of TGF-β signaling via the canonical pathway. ALK5, activin receptor-like kinase 5; ECM, extracellular matrix; EMT, epithelial-to-mesenchymal transition; EndMT, endothelial-to-mesenchymal transition; TβRI, TGF-β receptor type I; TβRII, TGF-β receptor type II.

The synthesis, release, and activation of TGF-β is a complex process (Figure 3). Following intracellular biosynthesis, a dimer of TGF-β is secreted as an inactive protein complex (latent TGF-β), which is retained in the ECM. Active TGF-β1 can be liberated from ECM by multiple activators such as reactive oxygen species (ROS), plasmin, thrombospondin-1, and αvβ6 integrin (68). Once active TGF-β is released from ECM, it binds to transmembrane TGF-β receptor type II (TβRII) of a target cell. This receptor-ligand interaction induces serine/threonine kinase activity of TβRII for autophosphorylation (69). The canonical pathway of TGF-β signaling is initiated after phosphorylated TβRII forms a stable heteromeric complex with TGF-β receptor type I (TβRI), also known as activin receptor-like kinase 5 (ALK5), for the transphosphorylation of residual phosphate to TβRI (70). This receptor binding complex, which is a heterotetrameric combination between two molecules of TβRII and another two of TβRI, recruits and phosphorylates the downstream signaling proteins Smad2 or Smad3, which are called receptor-activated Smads. After phosphorylation, Smad2 or Smad3 is released and forms an intracellular complex with Smad4, the mediator Smad. This intracellular complex between Smad2/4 or Smad3/4 moves from the cytoplasm into the nucleus, where it binds to promoter regions of the genes involved in physiological process of induction of specific gene expression (71). For an example of fibrogenesis, gene encoding α-SMA, collagens, and fibronectin are significantly upregulated via the Smad3-dependent pathway (72). The expression of these fibrosis-related genes plays a pivotal role in the cellular transdifferentiation that generates myofibroblasts and the production/deposition of ECM by myofibroblasts in fibrotic tissue (72). In addition to fibrogenesis, the Smad-mediated signaling pathway is also a significant intracellular process activated by TGF-β that increases genes associated with carcinogenesis (73). Furthermore, the activation of TGF-β signaling results in the expression of Smad7, an inhibitory SMAD, which acts as a negative regulator by interacting with Smad2 or Smad3, thereby mitigating signaling through receptor-activated Smads and further decreasing TGF-β actions (74).

**Figure 4:**
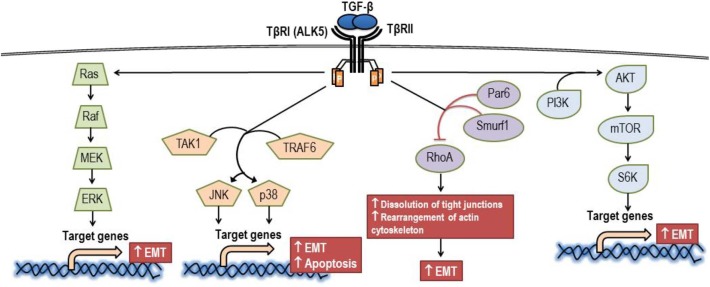
Signaling via the non-canonical pathway of TGF-β. AKT, protein kinase B; ALK5, activin receptor-like kinase 5; EMT, epithelial to mesenchymal transition; ERK, extracellular signal-regulated kinase; JNK, c-Jun amino terminal kinase; MEK, mitogen-activated protein kinase kinase; mTOR, mammalian target of rapamycin; Par6, partitioning-defective 6; PI3K, phosphoinositide 3-kinase; p38, p38 mitogen-activated protein kinase; Raf, Raf proto-oncogene serine/threonine-protein kinase; Ras, Ras GTPase; RhoA, Ras homolog gene family member A; Smurf1, SMAD specific E3 ubiquitin protein ligase; S6K, phosphorylation of S6 kinase; TAK1, TGF-β-activated kinase 1; TRAF6, tumor necrosis factor receptor-associated factor 6; TβRI, TGF-β receptor type I; TβRII, TGF-β receptor type II.

Beyond canonical pathways or Smad-mediated signaling, TGF-β might mediate signaling directly by activating kinase enzymes via non-Smad signaling pathways, which are also known as non-canonical pathways (Figure 4). The non-Smad signaling pathways are initially propagated by either or both phosphorylated TβRI and TβRII for modulating downstream cellular responses. It has been reported that crosstalk between canonical and non-canonical pathways appeared to occur in most TGF-β-mediated effects (75). Epithelial-to-mesenchymal transition (EMT) plays a significant role in the pathogenesis of cancer. In part, this process requires an activation of ERK by TGF-β to upregulate the genes involving in remodeling of cell-matrix adhesion, thereby promoting the motility of the transformed cells (76). Also, EMT might be induced by TGF-β via both TβRI and TβRII through the activation of TNF receptor-associated factor 6 (TRAF6). TRAF6 is capable of recruiting TGF-β-activated kinase 1 (TAK1) to subsequently allow the activation of c-Jun amino terminal kinase (JNK) and p38 mitogen-activated protein kinase (p38-MAPK) (77). In addition, the TRAF6-TAK1-JNK/p38 pathway is believed to be an essential pathway for TGF-β-induced apoptosis (78). Similar to the ERK and JNK/p38-MAPK pathway, the Ras homolog gene family member A (RhoA) is also a signaling mediator of EMT. TGF-β-induce RhoA degradation by phosphorylating partitioning-defective 6 (Par6), which subsequently recruits Smad-specific E3 ubiquitin protein ligase (Smurf1) to loosen tight junctions and rearrange the actin cytoskeleton, a prerequisite step for EMT (79). Another non-Smad signaling pathway contributing to TGF-β-promoted EMT is the phosphoinositide 3-kinase (PI3K)/Akt (protein kinase B) pathway, which subsequently activates the mammalian target of rapamycin (mTOR) and phosphorylation of S6 kinase (S6K) (80, 81). In addition, TGF-β1 signaling can be regulated at the post-transcriptional level via the expression of microRNAs (miRNAs), and the expression of miRNAs might play a role in TGF-β1-mediated EMT also (82).

### TGF-β Signaling in the Development of Cancers

For the ultimate outcome of TGF-β-mediated responses in any pathological condition, it is apparent that a combination of canonical and non-canonical pathways are coordinated (1). Cancers and fibrotic diseases are the most common pathologies associated with the activity of TGF-β. Currently, most putative drugs affecting TGF-β for the treatment of cardiac fibrosis were initially developed for the management of cancer; therefore, we next discuss the signaling of TGF-β in carcinogenesis.

In the pathogenesis of cancer, TGF-β acts as a tumor suppressor in early stages of the disease. However, in later stages, TGF-β turns into a tumor promoter. This paradoxical role of TGF-β is due to a bypass of the cytostatic effect of TGF-β in tumor cells (4). The tumor suppressive effect of TGF-β is derived from various cellular effects. TGF-β stabilizes the cell cycle of epithelial cells by upregulating multiple cyclin-dependent kinases: p15, p21, and p27, via the canonical pathway (83). Also, via the Smad-dependent pathway, TGF-β downregulates genes associated with cell proliferation, such as c-Myc (84). In addition, the canonical pathway contributes to the tumor suppressive effects of TGF-β by inducing gene encoding B-cell lymphoma 2 (BCL2) and subsequently activating BIM for apoptotic processes in human B cells (85). Conversely, non-canonical pathways might mediate the apoptotic effect of TGF-β by inducing caspase-8 expression and activating BID in human gastric carcinoma cells (86). The difference in signaling of TGF-β-mediated apoptosis indicates that the cellular context is essential for controlling the main pathway in the tumor suppressive effects of TGF-β. The tumor promoting effects of TGF-β such as EMT, invasion, metastasis, and angiogenesis emerge when cancer progresses to a later stage (5, 87). The upregulation of miR-106b-25 cluster targets Smad7 to ameliorate the TGF-β signaling that is not generally found in normal tissues is an excellent example of this phenomenon. In human breast cancer, increased miR-106b-25 leads to the inhibition of tumor suppressive protein p21 and BIM, thereby allowing tumor cells to grow via the activation of TGF-β (88). Interestingly, TGF-β also regulates the functions of various immune cells, including the modulation of cytokines released from these cells. Impairment of TGF-β signaling pathways leads to immune dysregulation, fibrosis, and cancer [Reviewed in (7)]. TGF-β is produced as a complex with latency associated peptide (LAP). This complex associates with ECM by binding to latent TGF-β binding protein (LTBP) or glycoprotein A repetitions predominant (GARP) expressed on T cells, especially on Tregs, or platelets. Integrins bind to the complex and stimulate the release of TGF-β from the complex. The release of active TGF-β promotes oncogenesis and immune tolerance in breast cancer (89). Inhibition of αvβ8 integrins potentiates cytotoxic T cell responses and recruitment of immune cells to tumor centers. Cancer cells can evade host immunity by mobilizing active TGF-β1 through αvβ8 integrins (90). Thus, TGF-β acts as a significant suppressor of immune responses during tumor progression.

In general, tissue fibrosis is considered a main step in triggering cancer development. An apparent example is hepatocellular carcinoma, the most common form of liver cancer. Cirrhosis, which is known as the end-stage of liver fibrosis, occurs in most patients who ultimately develop hepatocellular carcinoma (91). Interestingly, the progression of fibrosis to cancer in the heart is rare. The low incidence of cardiac cancer might be due to the fact that cardiac cells, in particular cardiomyocytes, are fully differentiated cells. Moreover, the regenerative capacity of cardiomyocytes is considered to be negligibly low. Thus, cardiomyocytes appear to resist further transformation and proliferation processes such as EMT in the development of cancer (92). Accordingly, signaling of TGF-β in fibrogenesis of the heart might not be identical to that occurring in other organs where progressive fibrosis ultimately develops cancers.

### TGF-β Signaling in the Development of Cardiac Fibrosis

During tissue injury, TGF-β expression is increased to play a role in the tissue repair process and scar formation. In the heart tissue following MI, TGF-β signaling plays an important role in reparative, angiogenetic, and fibrotic responses by modulating inflammation (93). Studies on mice and dogs have revealed that TGF-β1 and TGF-β2 were upregulated in the early phase after MI, and then TGF-β3 was increased in a later stage post-infarction myocardium (94). Among various cells that release TGF-β, a significant amount of TGF-β might be released from infiltrated macrophages that migrate to the injured area to engulf the damaged cardiomyocytes, as shown in a mouse model (95). On the other hand, a study using a porcine model of chronic coronary constriction revealed that cardiomyocytes were a significant source of TGF-β (96). Another study suggested that TGF-β was found in the extracellular fluid of ischemic canine myocardium tissue (97). Multiple pathways involving integrins and thrombospondin-1 were found to be associated with the release of TGF-β from the cardiac ECM-bound TGF-β (98, 99). Following the release of active TGF-β, TGF-β binds to the receptors, as described earlier, to activate intracellular responses in the infarcted tissue. The TGF-β-mediated effects can be classified into 4 actions in the following order: cardiomyocyte survival, immune cell-related action, formation of myofibroblasts, and production/deposition of ECM, all of which modulate the effects on myocardial endothelial cells.

TGF-β-mediated effects on cardiomyocyte survival in MI appear to be dependent on the time period after MI. In the early phase, exogenous TGF-β administered before or immediately after ischemic injury to an isolated perfused heart showed cardioprotective effects by reducing the amount of superoxide anions, maintaining coronary relaxation, and reducing injurious responses of exogenous TNF-α (100). Similarly, a study has shown that the infarct size of intact rat hearts receiving TGF-β during early reperfusion was reduced, and this reduction was due to activation of MAPK (101). However, the mechanism underlying cardioprotection remains poorly understood. Conversely, a proapoptotic effect of TGF-β via interplay with Ang II was demonstrated in a study using rat cardiomyocytes (102). The findings showed that the actions of exogenous TGF-β are likely dependent on the timing of administration.

Immune cells play a pivotal role in fibrogenesis, and TGF-β regulates both the phenotype and function of the immune cells. It is worth noting that TGF-β can be either a pro- or anti-inflammatory mediator of the immune response in *in vitro* studies [Reviewed in (93)]. Factors that determine the effects of TGF-β include the types of cytokines and the origin of the tissue (103). In an *in viv*o study, TGF-β suppressed T cell-mediated inflammation in genetically modified mice with T cell-specific loss of TβRII. Thus, the results from this *in vivo* study implicate an immunosuppressive effect of TGF-β (104). Nevertheless, the specific TGF-β-mediated effects on the phenotype of immune cells, together with its signaling and significance in the regulation of fibrosis, in the infarcted tissue remain unknown in the infarcted tissue.

TGF-β-mediated effects on the formation of myofibroblasts and on the induction of transformed myofibroblasts to further produce/deposit ECM are currently recognized central to the role of TGF-β in the pathogenesis of fibrosis. In cardiac fibrosis, Smad3-deficient mice that underwent reperfused MI showed significantly less fibroblast proliferation and ECM when compared to those of wild-type mice (105, 106). Even though the origin of the cells that underwent transformation has been debated (107), a recent study using fibroblast-specific, TGF-β signaling pathway knockout mice demonstrated that myofibroblasts in cardiac fibrosis are derived from resident fibroblasts, which activated via the TGF-β-Smad2/3 signaling pathway (72). These results suggest that the canonical pathway of TGF-β is principally involved in the pathogenesis of cardiac fibrosis. Interestingly, it was found that the Smad3-dependent pathway is essential for the upregulation of connective tissue growth factor (CTGF), which in turn acts as a mediator to stimulate fibroblast differentiation and collagen synthesis (108). Beyond the formation of myofibroblasts, genes encoding collagen type I and III were upregulated in cardiac fibroblasts isolated from rabbit hearts following treatment with TGF-β (109). The TAK1/p38-MAPK pathway in the cardiomyocytes of non-infarcted myocardium was found to be activated in rats after acute MI, suggesting a role for this non-canonical pathway in ventricular hypertrophy and remodeling (110). Nevertheless, the significance of Smad-independent pathways in the transformation of cardiac fibroblasts appears to be less proven than that of renal and pulmonary fibrosis (111, 112). Finally, a study on TGF-β-overexpressed mice showed increase expression of tissue inhibitors of matrix metalloproteinases (TIMPs), which regulate the remodeling of ECM in the cardiac tissue. However, the signaling of TGF-β was not evaluated in this study (113).

In addition to cardiomyocytes, immune cells, and transformed myofibroblasts, vascular endothelial cells might also play an important role in cardiac fibrosis. It has been found that endothelial cells served as a source of chemokines and played a role in recruiting neutrophils and monocytes to the heart after MI (114). Interestingly, although TGF-β plays a role in angiogenesis in cancers (8), information on the effects of TGF-β on angiogenesis in infarcted myocardium is limited at present. Moreover, although most cardiac myofibroblasts originate from resident fibroblasts, a study has shown that endothelial cells might be activated by the TGF-β via Smad3-dependent pathway and transform into myofibroblasts, thereby inducing cardiac fibrosis (115).

## TGF-β Inhibitors for the Treatment of Cancers and Cardiac Fibrosis

### Inhibitors of TGF-β Signaling for the Treatment of Cancers

TGF-β suppresses cell proliferation leading to apoptosis in the early phase of tumor development, whereas it aggravates tumor invasion and metastasis via boosting immune escape, angiogenesis, and EMT of tumors at an advanced stage (116). The paradoxical impact of TGF-β signaling in various tumors raises concerns that anti-TGF-β signaling might lead to a poor prognosis due to its tumor suppressor role. This concern has delayed progression in the development of TGF-β inhibitors as therapeutic agents. In addition, some experimental models have revealed that TβRI inhibitors aggravated the potential for cardiotoxicity (117).

However, several potential approaches to interfering with TGF-β signaling to prevent TGF-β production and block its signaling pathway have emerged. Next, we summarize the results of TGF-β inhibitors that have been studied in preclinical or clinical trials on carcinogenesis. The studies can be mainly categorized into 3 levels: (1) The ligand level: Direct blockage of TGF-β ligand synthesis by antisense molecules; (2) The ligand-receptor level: Inhibition of TGF-β ligand-receptor interaction using monoclonal antibodies or soluble TGF-β decoy receptors (traps); and (3) The intracellular level: Suppression of the TGF-β signaling pathway by tyrosine kinase inhibitors that disturb the downstream signaling of TGF-β related proteins (9, 118). The examples of current therapeutic agents in preclinical and clinical development in oncology are summarized in Tables 3, 4.

**Table 3:** Preclinical studies of TGF-β inhibitors for cancer treatment.

**Agents**	**Target**	**Experiments/Models**	**References**
1. THE LIGAND LEVEL			
Trabedersen (AP12009)	TGF-β2 mRNA	*In vivo*: patient-derived gliomas	(119)
		*In vivo*: induced melanoma tumor in mice	(120)
		*In vitro*: pancreatic carcinomas	(121)
		*In vivo*: human metastatic pancreatic cancer	(122)
		*In vivo*: human colon carcinomas	(123)
2. THE LIGAND-RECEPTOR LEVEL			
Soluble TβRII	TβRII	*In vitro*: human metastatic pancreatic cancer cells	(124)
		*In vivo*: patient-derived endometrial cancer	(125)
Soluble TβRIII (βglycan)	TβRIII	*In vivo*: patient-derived tissue from renal cancer	(126)
		*In vivo*: patient-derived tissue non-small-cell lung carcinoma	(127)
		*In vivo*: human xenograft model of breast cancer	(128)
3. THE INTRACELLULAR LEVEL			
Galunisertib (LY2157299)	TβRI	*In vivo*: patient-derived pancreatic, lung, colorectal cancer	(129)
		*In vivo*: human ovarian cancer in nude mice	(130)
		*In vitro*: hepatocellular carcinoma cells	(131–133)
Vactosertib (EW-7197)	TβRI	*In vivo*: lung metastases from breast cancer mice or transgenic MMTV/cNeu mice	(134)
EW-7195	TβRI	*In vivo*: lung metastases from breast cancer mice	(135)
LY2109761	TβRI/II	*In vivo*: metastatic colorectal cancer	(136)
		*In vivo*: metastatic hepatocellular carcinoma	(137)
SD208	TβRI	*In vivo*: metastatic breast cancer	(138)
		*In vivo*: metastatic pancreatic cancer	(139)

**Table 4:** Clinical studies of TGF-β inhibitors for cancer treatment.

**Agents**	**Target**	**Phase**	**Study design**	**Main findings**	**References**
1. THE LIGAND LEVEL					
Trabedersen (AP12009)	TGF-β2 mRNA	IIb	A randomized controlled trial compared to standard chemotherapy in refractory malignant (high-grade) glioma (*N* = 145)	Unchanged tumor growth Delayed responses after treatment discontinuation	(140)
2. THE LIGAND-RECEPTOR LEVEL					
Belagenpumatucel-L	TGF-β2	II	A randomized, dose-variable trial in stages II, IIIA, IIIB, and IV non-small-cell lung carcinoma (NSCLC) (*N* = 75)	Improved overall survival (OS) Increased IFN-γ, IL-4, and IL-6 production	(141)
Belagenpumatucel-L	TGF-β2	II	A randomized trial in advanced NSCLC (*N* = 21)	Increased OS	(142)
Belagenpumatucel-L	TGF-β2	III	A randomized trial in stage III/IV NSCLC after platinum-based therapy (*N* = 532)	Unchanged OS	(143)
Fresolimumab (GC-1008)	Pan TGF-β	II	An open-label trial in malignant pleural mesothelioma (*N* = 13)	Increased OS in patients who produced antitumor antibodies	(144)
Fresolimumab (GC-1008)	Pan TGF-β	II	An open label randomized trial in metastatic breast cancer with radiotherapy (*N* = 23)	Increased OS Well-tolerated Higher dose improved CD8	(145)
3. THE INTRACELLULAR LEVEL					
Galunisertib (LY2157299)	TβRI	II	A randomized study in metastatic pancreatic adenocarcinoma used gemcitabine for first-line therapy (*N* = 156)	Improved OS	(146)
Galunisertib (LY2157299)	TβRI	II	A randomized trial in hepatocellular carcinoma treated with galunisertib as monotherapy after sorafenib failure (*N* = 109)	Median OS of 8.3 months	(147)
Tasisulam (LY573636)	TGF-β	II	A randomized study as second-line or third-line treatment for metastatic soft tissue sarcoma (*N* = 101)	Modest activity as second-/third-line treatment (Median OS = 8.71 months)	(148)

#### Trabedersen (AP12009)

##### Preclinical data

Trabedersen (AP12009, Antisense Pharma) is a synthetic, 18-oligomer phosphorothioate antisense oligonucleotide (ASO). It was developed as an ASO specifically targeting human TGF-β2 mRNA, which leads to a reduction in TGF-β2 expression, cellular proliferation, and cellular migration in various types of tumors *in vitro* and *in vivo*, including gliomas (119), melanoma (120), pancreatic carcinomas (121, 122), and colorectal cancer (123). Trabedersen has been shown to reduce cell proliferation, tumor growth, cell migration or metastasis, and vascularization in human pancreatic cancer cells and in mouse model of human metastatic pancreatic cancer (122).

##### Clinical data

After several preclinical studies provided evidence of potential clinical efficacy, trabedersen was moved to phase I/II trials in patients with recurrent high-grade gliomas (119, 140, 149). Trabedersen was initially assessed for its safety and efficacy in phase I/II dose escalation studies in patients with high-grade gliomas and found a significant increase of median survival time after recurrence, exceeding that of standard chemotherapy (149). Similarly, prolonged survival and high response rates after treatment with trabedersen were observed in phase I/II studies in patients with recurrent or refractory malignant glioma, WHO grade III or IV (119). However, trabedersen was further compared with standard chemotherapy (temozolomide or procarbazine/lomustine/vincristine) in patients with recurrent or refractory malignant glioma (WHO grade III or IV) in a phase IIb trial. The results revealed that trabedersen did not control tumor growth, but delayed responses were observed after discontinuation of treatment (140).

#### Belagenpumatucel-L Vaccine

The principle of anti-TGF-β cancer vaccines is to deliver antisense molecules of TGF-β into cancer cells and overturn the effects of immunosuppression in host cells, as well as to enhance antitumor immunity (9). Belagenpneumatucel–L (Lucanix, NovaRx) is a TGF-β2, antisense, gene-modified non-viral based allogenic tumor cell vaccine. It was developed from non-small cell lung cancer (NSCLC) and modified to express ASO, which leads to suppression of the immunosuppressive activity implicit in TGF-β2 overexpressing cancer cells (141).

##### Clinical data

Currently, an anti-TGF-β cancer vaccine, belagenpumatucel-L, has entered a phase III study to determine whether it improves overall survival (OS) and might be useful for stimulating immune reactions. A dose-related survival difference was achieved in patients who received belagenpumatucel-L at least 2.5 × 10^7^ cells/injection in a phase II trial involving patients with stages II, III, and IV NSCLC. Moreover, immune function measurements revealed an increase in cytokine production, including IFN-γ, IL-6, and IL-4, among clinical responders, who also displayed an elevated antibody-mediated response to the vaccine human leukocyte antigens (HLAs) (141). Likewise, a further study to evaluate its safety and response at the previously defined optimal dose found the median survival of patients with fewer than 2 circulating tumor cells (CTCs) at baseline was longer than patients with 2 or more CTCs. Thus, plasma levels of CTCs are associated with the OS of patients with stage IV NSCLC (142). Nevertheless, in a phase III trial with 532 patients with stage III/IV NSCLC who did not progress after platinum-based induction chemotherapy with or without irradiation, belagenpumatucel-L did not increase survival compared with placebo (143).

#### Fresolimumab (GC1008)

##### Clinical Data

Fresolimumab (GC1008, Genzyme/Sanofi) is a fully human monoclonal antibody blocking pan-TGF-β (TGF-β1, TGF-β2, and TGF-β3) [Reviewed in (150)]. Fresolimumab demonstrated acceptable safety and preliminary evidence of antitumor activity in a phase I trial on patients with previously treated malignant melanoma or renal cell carcinoma (151). In a phase II trial on 13 patients with malignant pleural mesothelioma, 3 patients showed stable disease for at least 3 months, and those who produced antitumor antibodies had an increased median OS. However, treatment with fresolimumab had no effect on the expression of NK, CD4^+^, or CD8^+^ T cell activating and inhibitory markers, other than a decrease in the expression of CD244 (also known as 2B4) and CD266 (best known as DNAM1) on NK cells (144). A phase II trial on 23 patients with metastatic breast cancer undergoing radiotherapy has reported that fresolimumab in combination with focal radiotherapy significantly increased OS and was well-tolerated in a dose-dependent manner. Higher doses of fresolimumab correlated with an improved CD8^+^ pool, leading to a favorable systemic immune response and longer median OS (145).

#### Galunisertib (LY2157299)

##### Preclinical Data

Galunisertib monohydrate (LY2157299, Eli Lilly) is a small-molecule inhibitor of TβRI that robustly downregulate the phosphorylation of Smad2 in pancreatic, lung, colorectal (129), and ovarian cancer (130). Galunisertib effectively demonstrated potent inhibition of both canonical and non-canonical pathways in a variety of *in vitro* hepatocellular carcinoma cells regardless of TGF-β pathway protein expression (131, 132). Nevertheless, the antiproliferative activity of TGF-β pathway inhibitors is quite limited. It has been reported that TGF-β inhibited cell proliferation while inducing apoptosis in cell lines with low endogenous levels of TGF-β and Smad7 and strong transcriptional Smad3 activity (PLC/PRF/5, HepG2, Hep3B, HuH7). However, cancer cells were sensitive to TGF-β-dependent growth inhibition and displayed limited sensitivity to galunisertib in another group of cell lines expressing high quantities of TGF-β and Smad7 and showing significantly reduced Smad3 signaling (SK-HEP1, SK-Suni, SK-Sora, JHH6, HLE, HLF, and FLC-4) (132, 133). Despite limited antiproliferative activity *in vitro*, galunisertib exhibited antiproliferative effects in *ex vivo* models, indicating that inhibition of TGF-β can exert anticancer properties (131, 133). Nevertheless, from the reports on several preclinical studies, treatment with TGF-β inhibitors as monotherapy might display limited efficacy. However, the immunological effects of galunisertib are strongly augmented in combination with other checkpoint inhibitors (152, 153).

##### Clinical data

Among small molecule inhibitors, galunisertib is one of the most advanced. It has shown promising results in clinical trials due to its safety profile, with no cardiac potential toxicity in humans, which was a primary concern with first-generation TGF-β inhibitors (154). A phase I study on 28 patients with Grade IV glioma showed galunisertib was well-tolerated. The dose limiting toxicities included pulmonary embolism and thrombocytopenia, but no cardiotoxicities were observed (155). In addition, the safety of galunisertib was confirmed by a first-in-human dose study with 79 cancer patients with glioma and solid tumors treated with galunisertib as monotherapy or in combination with lomustine. No medically relevant cardiac toxicity or signs of cardiovascular injury were found, including increased blood pressure, troponin I, BNP, or hs-CRP or reductions in cystatin C levels (156). Likewise, no safety concerns or dose limiting toxicities was observed after treatment with galunisertib in patients with glioblastoma based on a pharmacokinetic/pharmacodynamic (PK/PD) model (157). Galunisertib as monotherapy and as second-line therapy after sorafenib failure in a subset of 109 patients with hepatocellular carcinoma yielded a median OS of 8.3 months in a phase II trial (147). Interestingly, patients who had decreased expression levels of specified blood biomarkers [e.g., alpha-fetoprotein (AFP), TGF-β1, and CDH1] had improved clinical outcomes, indicating that the effects of galunisertib might be more pronounced in patients with a poor prognosis due to elevated AFP at baseline (147). Similarly, galunisertib in combination with gemcitabine improved OS with minimal added toxicity in a phase II study on patients with locally advanced or metastatic pancreatic adenocarcinoma who were considered candidates for first-line chemotherapy with gemcitabine (146).

#### Vactosertib (EW-7197) and EW-7195

##### Preclinical Data

Vactosertib (EW-7197 or TEW-7197), a novel small molecule inhibitor of ALK5, has been recently developed as a more potent and specific antitumoral compound than galunisertib. Vactosertib and EW-7195 expressed potent antimetastatic activity *in vivo* via an inhibition of TGF-β1-induced Smad/TGFβ signaling, cell migration, invasion, EMT, and breast tumor metastasis to the lung in xenografted nude mice and transgenic MMTV/cNeu mice (134, 135). In addition, vactosertib expressed the potential to boost cytotoxic T lymphocyte function in 4T1 orthotopic-grafted mice and prolonged the lifespan of 4T1 breast tumor-bearing mice (134).

##### Clinical data

Vactosertib is currently being tested in phase I/II clinical trials for several cancer types in combination with chemotherapy or antibodies against immune checkpoints. A phase I study is evaluating the safety and tolerability of the drug in combination with paclitaxel in 12 metastatic gastric cancer patients (NCT03698825). The phase Ib/IIa trials include a study of vactosertib in combination with durvalumab in patients with advanced NSCLC who progressed following platinum-based chemotherapy (*N* = 63) (NCT03732274). A combination with pembrolizumab is being employed for metastatic or locally advanced colorectal or gastric/gastroesophageal junction adenocarcinoma (*N* = 67) (NCT03724851), and a combination with imatinib is being employed for patients with advanced desmoid tumors (*N* = 24) (NCT03802084). The latest phase II trial aims to determine whether administration of vactosertib with durvalumab will provide meaningful increases in the overall response rate in patients with urothelial cancers that fail to achieve a CR with anti-PD-1/PD-L1 based regimens (*N* = 48) (NCT04064190).

Remarkably, given TGF-β signaling plays a crucial role in fibrotic states, vactosertib has recently been investigated as an antifibrotic agent to delay the development of fibrosis in primary organs including the liver, kidney, and lung. Vactosertib was found to suppress fibrosis-induced accumulation of ROS and ECM proteins (collagen, α-SMA, fibronectin, and integrins) in the liver, lungs, and kidneys of mice due to its antifibrotic mechanism via inhibition of both TGF-β1/Smad2/3 and ROS signaling (158). A study on a rat model of Peyronie's disease showed that vactosertib suppressed phospho-Smad2 expression and recruitment of inflammatory cells, leading to a decline in fibrotic plaques (159). Thus, vactosertib and EW-7195 could be a promising antifibrotic compound for the treatment of fibrotic diseases.

#### Tasisulam (LY573636)

##### Clinical Data

Tasisulam has completed many trials in various oncologic diseases, including phase I studies on patients with essential thrombocythemia and acute myeloid leukemia (NCT00718159) and solid tumors (NCT01214668) and phase II trials on patients with ovarian cancer (NCT00428610), metastatic breast cancer (NCT00992225), NSCL cancer (NCT00363766), and malignant melanoma (NCT00383292). A phase II study on tasisulam as second- or third-line treatment for 101 patients with unresectable or metastatic soft tissue sarcoma reported that tasisulam demonstrated modest activity with a median OS of 8.71 months (148). Consequently, the synergistic and additive effects of tasisulam combined with other anticancer agents are currently of interest. Currently there is an ongoing phase I trial of tasisulam in combination with sunitinib, a multiple tyrosine kinase, in renal cancer patients (NCT01258348), and with pemetrexed, an inhibitor of purine synthesis, in patients with solid tumors (NCT01215916).

##### M7824 (MSB0011359C)

Interestingly, recent preclinical study has been reported that M7824 (MSB0011359C) which is a dual inhibitor of programmed death ligand 1 (PD-L1) and TGF-β inhibited tumor growth and metastasis more effectively than treatment with TGF-β inhibitor alone. Thus, M7824 (an inhibitor of PD-L1 and TGF-β) exhibits potent and superior antitumor effects compared to that of TGF-β inhibitor monotherapy and is likely to help minimize potential side effects (160).

### Inhibitors of TGF-β Signaling for the Treatment of Cardiac Fibrosis

The renin-angiotensin system (RAS) inhibitors are currently used as standard therapy for HF and have been shown to inhibit activation of fibroblast and differentiation into myofibroblast. However, cardiac fibrosis persists in patients with HF even when treated with these conventional RAS inhibitors, indicating a need to develop novel and effective antifibrotic therapies for heart disease (161). Currently, due to its established role in cardiac fibrosis, there is great interest in inhibiting the TGF-β signaling pathway (6, 161). TGF-β is considered a mediator of cancer and fibrosis. Thus, blockades of TGF-β signaling activity using receptor antagonists, inhibition via antibody or antisense oligonucleotide, or even using gene deletion of TGF-β signaling molecules are potential therapeutic strategies.

Anti-TGF-β1 neutralizing antibodies have also been under investigation as potential antifibrotic agents by interfering with TGF-β signaling. Administration with anti-TGF-β1 antibody attenuated cardiac fibrosis and diastolic abnormalities in a rat model of pressure overload (47) (Table 2). Although these antibodies attenuated fibroblast activation and collagen synthesis, no improvements in overall cardiac functions were found in pressure-overloaded rats (47). Furthermore, anti-TGF-β neutralizing antibody inhibited ECM proteins synthesis and reduced cardiac fibrosis in a rat model induced by a chronic blockade of nitric oxide synthesis (162). However, in a mouse model of MI, a neutralizing anti-TGF antibody administered before or after coronary artery ligation resulted in increased mortality rates and left ventricular (LV) dilation after MI (163).

Alternative approaches have included inhibition of the expression of TGF-β using antisense oligonucleotides (164), and the use of a soluble TβRII, which either acts by adsorbing TGF-β or acting as a dominant negative receptor (165). Inhibitors of ALK5 (TβRI) are under investigation for antifibrotic effects in the heart. Inhibitor of ALK5 which decrease TGF-β activity can rescue cardiac dysfunction and ameliorate cardiac remodeling in post-MI hearts (50). Moreover, ALK5 inhibitors can also suppress the collagen synthesis and attenuate the progression of fibrosis in animal model of pressure overload induced by transverse aortic constriction, and inhibit TGF-β-mediated collagen synthesis in cardiac fibroblasts (51) (Table 2).

In addition to the canonical Smad-mediated signaling pathway, TGF-β also stimulates the non-canonical MAPK signaling pathways such as JNK-dependent and p38-MAPK-dependent pathways (166–168). These MAPK signaling pathways are involved in TGF-β-mediated activation of TAK1 which is thought to play a role in cardiac fibrosis and remodeling. Cardiac specific overexpression of the active form of TAK1 induced myocardial hypertrophy and HF (166–168), suggesting that TAK1 is a major effector of TGF-β signaling. Blockade of TAK1 activity attenuated TGF-β-mediated ECM protein overproduction in cardiac fibroblasts (48) (Table 2). In addition to inhibition of TAK1, inhibition of p38-MAPK is being investigated for its efficacy in the treatment of cardiac fibrosis. Inhibitors of p38-MAPK suppress myofibroblast activation and expression of ECM proteins and α-SMA induced by TGF-β, while overexpression of p38-MAPK induces myofibroblast differentiation in cardiac fibroblasts (49).

Two promising antifibrotic agents include tranilast and pirfenidone, which inhibit the actions of TGF-β as well as other pathogenic growth factors by unclear mechanisms (169). Current agents and therapeutic targets in preclinical and clinical development for the treatment of cardiac fibrosis and heart-related diseases are summarized in Tables 5, 6.

**Table 5:** Preclinical studies of TGF-β inhibitors for treatment of cardiac fibrosis.

**Agents**	**Targets**	**Experiments/Models**	**References**
GW788388	ALK5 and TβRII	*In vivo*: murine Chagas disease	(170)
		*In vivo*: *Scn5a^+/−^* mouse model of cardiac conduction disease	(171)
		*In vivo*: rat model of heart failure (HF) following myocardial infarction (MI)	(50)
Pirfenidone	TGF-β	*In vivo*: Deoxycorticosterone acetate (DOCA)-salt hypertensive rats	(172)
		*In vivo*: rat MI model	(173)
		*In vivo*: Transverse aortic constriction (TAC)-induced left ventricular (LV) remodeling mouse model	(174)
		*In vivo*: TAC-induced pressure-overloaded HF model	(175)
		*In vivo*: Streptozotocin (STZ)-induced diabetic rats	(176)
Tranilast	TGF-β	*In vivo*: STZ-induced diabetic (mRen2)27 rats	(177, 178)
		*In vivo*: DOCA/salt and renovascular hypertensive rats	(179, 180)
		*In vivo*: LV remodeling post-MI rats	(181)
		*In vivo*: hypertensive (mRen2)27 rats	(182)

**Table 6:** Clinical studies of TGF-β inhibitors for treatment of cardiac fibrosis.

**Agents**	**Phase**	**Study design**	**Main findings**	**References**
Pirfenidone	II	A double-blind placebo-controlled phase II study in hypertrophic cardiomyopathy associated with left ventricular diastolic function patients (*N* = 50)	Not available	NCT00011076
Pirfenidone	II	A double-blind, randomized, placebo-controlled phase II trial in patients with chronic heart failure with preserved ejection fraction (HFpEF) and myocardial fibrosis (*N* = 129)	Not available	NCT02932566
Tranilast	III	A double-blind, randomized, placebo-controlled phase III trial in 11,484 patients after percutaneous coronary intervention (PCI) (PRESTO)	Tranilast did not improve the quantitative measures of restenosis	(183)

#### GW788388

##### Preclinical data

GW788388 is a potent inhibitor of both ALK5 and TGβRII with an improved pharmacokinetic profile (184) and minimal toxic effects (185). Several studies have been demonstrated that GW788388 pre-clinically reduces cardiac fibrosis in various models. GW788388 inhibited the development of cardiac fibrosis by suppression of collagen I and fibronectin synthesis, increased survival, and improved cardiac function in an experimental murine model of Chagas heart disease (170). Deletion of SCN5A, a gene encoding the main cardiac sodium channel NaV_1.5_, has been associated with inherited progressive cardiac conduction disease. GW788388 chronically inhibited TGF-β receptors and prevented fibrosis in a Scn5a heterozygous knockout (*Scn5a*^+/−^) mouse model of progressive cardiac conduction disease (171). Furthermore, treatment with GW788388 attenuated systolic dysfunction and delayed LV remodeling by reducing the phosphorylated Smad2, α-SMA, and collagen I in a rat model of HF following MI (50). Taken together, GW788388 appears to be a promising antifibrotic agent, although further studies are warranted.

#### Pirfenidone

##### Preclinical data

Pirfenidone is an oral antifibrotic drug initially approved for the treatment of idiopathic pulmonary fibrosis (186). Pirfenidone inhibited TGF-β expression and also inhibited the profibrotic effects of TGF-β signaling (187). Thus, pirfenidone might be a promising agent for the treatment of cardiac fibrosis. A reduction in ventricular hypertrophy without lowering systolic blood pressure has been detected in the deoxycorticosterone acetate (DOCA)-salt hypertensive rats after pirfenidone treatment (172). Moreover, pirfenidone decreased total and non-scar myocardial fibrosis, which has been associated with decreased infarct scarring, improved LV function, and decreased ventricular tachycardia in rat MI model (173). Administration of pirfenidone reversed cardiac fibrosis, including renal fibrosis, and attenuated myocardial stiffness in streptozotocin (STZ)-diabetic rats (176).

Given pirfenidone has significant antifibrotic and anti-inflammatory properties, the anti-inflammatory effects of pirfenidone have been investigated. Pirfenidone inhibited NLRP3 expression and formation, contributing to a reduction in IL-1β synthesis, and attenuation of IL-1β-induced inflammatory and profibrotic responses in a mouse model with transverse aortic constriction (TAC)-induced LV remodeling (174). Similar effects were observed in murine pressure-overload injury; pirfenidone increased survival and attenuated fibrosis through suppression of myocardial fibrosis and vascular permeability in pressure-overloaded hearts (175). Therefore, pirfenidone might be a potential treatment for cardiac fibrosis.

##### Clinical data

Although pirfenidone has shown efficacy in the treatment of idiopathic pulmonary fibrosis in humans (186), clinical trials for the treatment of cardiac fibrosis are ongoing and the results have not yet been published. A phase II study of pirfenidone in patients with hypertrophic cardiomyopathy associated with LV diastolic function aims to examine the effectiveness of pirfenidone in improving heart function and reducing of myocardial fibrosis. The study was completed with unpublished data (NCT00011076). Another phase II trial is ongoing and will finish in Jan 2020. This trial is exploring the antifibrotic effects of pirfenidone on patients with chronic heart failure with preserved ejection fraction (HFpEF) and cardiac fibrosis by determining changes in myocardial ECM volume and investigating the relationship between myocardial fibrosis and myocardial energetics (PIROUETTE study, NCT02932566) (188).

#### Tranilast

##### Preclinical data

Tranilast has been used to treat allergic disorders (e.g., allergic rhinitis, asthma, and atopic dermatitis); however, tranilast might also be useful for other medical conditions due to its ability to suppress TGF-β expression and activity. The molecular mechanisms underlying its antifibrotic actions are not completely understood, but tranilast might inhibit several profibrotic growth factors such as TGF-β and platelet-derived growth factor (PDGF) (22). The effects of tranilast on inhibition of cardiac fibrosis have also been supported by multiple animal models of cardiomyopathy. In STZ-induced (mRen-2)27 diabetic rats, tranilast treatment attenuated cardiac matrix deposition in association with reductions in phospho-Smad2 of the heart (177). In a similar model, administration of tranilast attenuated cardiac dysfunction and structural abnormalities in diabetic cardiomyopathy with improved LV systolic and diastolic function, while tranilast did not affect Smad phosphorylation but it significantly attenuated TGF-β-induced p44/42 MAPK phosphorylation (178).

The underlying mechanisms of the antifibrotic effects of tranilast have been attributed to its regulation of TGF-β signaling and to suppression of the infiltration of inflammatory cells, including monocytes and macrophages. The mRNA levels of TGF-β1, plasminogen activator inhibitor 1 (PAI-1), monocyte chemotactic protein-1 (MCP-1), IL-6, procollagens were attenuated, and myocardial fibrosis and collagen accumulation were suppressed in DOCA/salt hypertensive rats receiving tranilast (179). Similar findings were observed in other animal models of renovascular hypertensive rats (180) and hypertensive (mRen-2)27 rats (182). Interestingly, tranilast-mediated inhibition of cardiac fibrosis is independent of changes in blood pressure in these studies, suggesting that tranilast directly targeted cardiac fibrosis and might be beneficial for HF treatment in addition to current therapeutic strategies (181).

##### Clinical data

Restenosis after percutaneous coronary intervention (PCI) is a major adverse outcome following stent placement. In limited trials, administration of tranilast reduced the frequency of angiographic restenosis after PCI (189). Accordingly, the Prevention of Restenosis With Tranilast and Its Outcomes (PRESTO) trial was designed as a phase III trial with a large group of patients after PCI to investigate major adverse cardiovascular events of tranilast. It was found that tranilast did not improve restenosis or its clinical sequelae in patients receiving successful PCI (183). However, the number of events of MI was significantly reduced with tranilast treatment. The most commonly reported adverse events were laboratory test abnormalities consisting of hyperbilirubinemia, elevations in hepatic enzymes, and increased serum creatinine (183).

## Conclusion

TGF-β is a multifunctional cytokine regulator acting through transmembrane serine/threonine kinase receptors and intracellular Smad transcriptional regulators. Once TGF-β is activated, it regulates ECM remodeling and promotes a fibroblast to myofibroblast transition, which is essential for fibrotic processes. Given TGF-β plays a major role in various stages of cancer progression and in the development of cardiac fibrosis, TGF-β and its signaling pathway offer opportunities for novel treatment strategies in patients with cancer and cardiac fibrosis. Research on the underlying mechanisms and the therapeutic targets of TGF-β inhibitors for cancer and cardiac fibrosis has advanced significantly in recent decades. The inhibitors of TGF-β signaling for cancer and fibrosis have been extensively studied in animal models and clinical studies; however, translation of these findings into human pathologic conditions has been limited due to the broad range of responses to TGF-β and its role in tissue homeostasis. Currently, various types of TGF-β inhibitors are challenged and tested their efficacies in patients with cancers. A few of TGF-β inhibitors are subjected into the clinical studies for treatment of cardiac fibrosis. The development of more specific agents targeting TGF-β signaling pathways such as M7824, a bifunctional fusion protein composed of TGF-β trap, and a monoclonal antibody against programmed death ligand 1 (PD-L1) are likely to help minimize potential side effects and enhances efficacy for treatment of cancers. Furthermore, the combination of anti-TGF-β therapies with various mechanisms of action might have greater efficacy against cancer and cardiac fibrosis.

## Author Contributions

WP, TL, and SM wrote the manuscript. HK reviewed and edited. All authors agree to submit the manuscript.

### Conflict of Interest

The authors declare that the research was conducted in the absence of any commercial or financial relationships that could be construed as a potential conflict of interest.

## References

[1] Blobe GC Schiemann WP Lodish HF. Role of transforming growth factor-β in human disease. N Engl J Med. (2000) 342:1350–8. 10.1056/NEJM200005043421807 10793168

[2] Meng XM Nikolic-Paterson DJ Lan HY. TGF-β: the master regulator of fibrosis. Nat Rev Nephrol. (2016) 12:325–8. 10.1038/nrneph.2016.48 27108839

[3] Roberts AB. Molecular and cell biology of TGF-beta. Miner Electrolyte Metab. (1998) 24:111–9. 10.1159/000057358 9525693

[4] Smith AL Robin TP Ford HL. Molecular pathways: targeting the TGF-β pathway for cancer therapy. Clin Cancer Res. (2012) 18:4514–21. 10.1158/1078-0432.CCR-11-3224 22711703

[5] Xie F Ling L van Dam H Zhou F Zhang L. TGF-β signaling in cancer metastasis. Acta Biochim Biophys Sin. (2018) 50:121–32. 10.1093/abbs/gmx123 29190313

[6] Walton KL Johnson KE Harrison CA. Targeting TGF-β mediated SMAD signaling for the prevention of fibrosis. Front Pharmacol. (2017) 8:461. 10.3389/fphar.2017.00461 28769795 PMC5509761

[7] Batlle E Massagué J. Transforming growth factor-β signaling in immunity and cancer. Immunity. (2019) 50:924–40. 10.1016/j.immuni.2019.03.024 30995507 PMC7507121

[8] Pardali E ten Dijke P. Transforming growth factor-β signaling and tumor angiogenesis. Front Biosci. (2009) 14:4848–61. 10.2741/3573 19482591

[9] Haque S Morris JC. Transforming growth factor-β: a therapeutic target for cancer. Hum Vaccin Immunother. (2017) 13:1741–50. 10.1080/21645515.2017.1327107 28575585 PMC5557219

[10] Khan R Sheppard R. Fibrosis in heart disease: understanding the role of transforming growth factor-β in cardiomyopathy, valvular disease and arrhythmia. Immunology. (2006) 118:10–24. 10.1111/j.1365-2567.2006.02336.x 16630019 PMC1782267

[11] Biernacka A Dobaczewski M Frangogiannis NG. TGF-β signaling in fibrosis. Growth Fact. (2011) 29:196–202. 10.3109/08977194.2011.595714 21740331 PMC4408550

[12] Pohlers D Brenmoehl J Löffler I Müller CK Leipner C Schultze-Mosgau S. TGF-beta and fibrosis in different organs- molecular pathway imprints. Biochim Biophys Acta. (2009) 1792:746–56. 10.1016/j.bbadis.2009.06.004 19539753

[13] Najafi F Jamrozik K Dobson AJ. Understanding the 'epidemic of heart failure: a systematic review of trends in determinants of heart failure. Eur J Heart Fail. (2009) 11:472–9. 10.1093/eurjhf/hfp029 19251729

[14] Cohn JN Ferrari R Sharpe N. Cardiac remodeling- concepts and clinical implications: a consensus paper from an international forum on cardiac remodeling. J Am Coll Cardiol. (2000) 35:569–82. 10.1016/S0735-1097(99)00630-0 10716457

[15] Norton GR Tsotetsi J Trifunovic B Hartford C Candy GP Woodiwiss AJ. Myocardial stiffness is attributed to alterations in cross-linked collagen rather than total collagen or phenotypes in spontaneously hypertensive rats. Circulation. (1997) 96:1991–8. 10.1161/01.CIR.96.6.1991 9323091

[16] Liu J Masurekar MR Vatner DE Jyothirmayi GN Regan TJ Vatner SF. Glycation end-product cross-link breaker reduces collagen and improves cardiac function in aging diabetic heart. Am J Physiol Heart Circ Physiol. (2003) 285:H2587–91. 10.1152/ajpheart.00516.2003 12946933

[17] Yue Y Meng K Pu Y Zhang X. Transforming growth factor-β (TGF-β) mediates cardiac fibrosis and induces diabetic cardiomyopathy. Diabetes Res Clin Pract. (2017) 133:124–30. 10.1016/j.diabres.2017.08.018 28934669

[18] Voskoboinik A Costello BT La Gerche A Prabhu S Wong G Flannery MD. Relation of alcohol consumption to left ventricular fibrosis using cardiac magnetic resonance imaging. Am J Cardiol. (2019) 123:460–5. 10.1016/j.amjcard.2018.10.026 30473327

[19] El Hajj EC El Hajj MC Voloshenyuk TG Mouton AJ Khoutorova E Molina PE. Alcohol modulation of cardiac matrix metalloproteinases (MMPs) and tissue inhibitors of MMPs favors collagen accumulation. Alcohol Clin Exp Res. (2014) 38:448–56. 10.1111/acer.12239 24033327 PMC4080812

[20] Ma ZG Yuan YP Wu HM Zhang X Tang QZ. Cardiac fibrosis: new insights into the pathogenesis. Int J Biol Sci. (2018) 14:1645–57. 10.7150/ijbs.28103 30416379 PMC6216032

[21] Russo I Frangogiannis NG. Diabetes-associated cardiac fibrosis: cellular effectors, molecular mechanisms and therapeutic opportunities. J Mol Cell Cardiol. (2016) 90:84–93. 10.1016/j.yjmcc.2015.12.011 26705059 PMC4718740

[22] Edgley AJ Krum H Kelly DJ. Targeting fibrosis for the treatment of heart failure: a role for transforming growth factor-β. Cardiovasc Ther. (2012) 30:e30–40. 10.1111/j.1755-5922.2010.00228.x 21883991

[23] Kong P Christia P Frangogiannis NG. The pathogenesis of cardiac fibrosis. Cell Mol Life Sci. (2014) 71:549–74. 10.1007/s00018-013-1349-6 23649149 PMC3769482

[24] DeVita VT Jr Rosenberg SA. Two hundred years of cancer research. N Engl J Med. (2012) 366:2207–14. 10.1056/NEJMra1204479 22646510 PMC6293471

[25] Shewach DS Kuchta RD. Introduction to cancer chemotherapeutics. Chem Rev. (2009) 109:2859–61. 10.1021/cr900208x 19583428 PMC4110949

[26] Lumachi F Luisetto G Basso SM Basso U Brunello A Camozzi V. Endocrine therapy of breast cancer. Curr Med Chem. (2011) 18:513–22. 10.2174/092986711794480177 21143113

[27] Tammela TA. Endocrine treatment of prostate cancer. J Steroid Biochem Mol Biol. (2004) 92:287–95. 10.1016/j.jsbmb.2004.10.005 15663992

[28] Baudino TA. Targeted cancer therapy: the next generation of cancer treatment. Curr Drug Discov Technol. (2015) 12:3–20. 10.2174/1570163812666150602144310 26033233

[29] Zhang H Chen J. Current status and future directions of cancer immunotherapy. J Cancer. (2018) 9:1773–81. 10.7150/jca.24577 29805703 PMC5968765

[30] Mansoori B Mohammadi A Davudian S Shirjang S Baradaran B. The different mechanisms of cancer drug resistance: a brief review. Adv Pharm Bull. (2017) 7:339–48. 10.15171/apb.2017.041 29071215 PMC5651054

[31] Frangogiannis NG. Pathophysiology of myocardial infarction. Compr Physiol. (2015) 5:1841–75. 10.1002/cphy.c150006 26426469

[32] Kass DA Bronzwaer JG Paulus WJ. What mechanisms underlie diastolic dysfunction in heart failure? Circ Res. (2004) 94:1533–42. 10.1161/01.RES.0000129254.25507.d6 15217918

[33] Burlew BS Weber KT. Cardiac fibrosis as a cause of diastolic dysfunction. Herz. (2002) 27:92–8. 10.1007/s00059-002-2354-y 12025467

[34] López B Querejeta R González A Larman M Díez J. Collagen cross-linking but not collagen amount associates with elevated filling pressures in hypertensive patients with stage C heart failure: potential role of lysyl oxidase. Hypertension. (2012) 60:677–83. 10.1161/HYPERTENSIONAHA.112.196113 22824984

[35] Woodiwiss AJ Tsotetsi OJ Sprott S Lancaster EJ Mela T Chung ES. Reduction in myocardial collagen cross-linking parallels left ventricular dilatation in rat models of systolic chamber dysfunction. Circulation. (2001) 103:155–60. 10.1161/01.CIR.103.1.155 11136701

[36] Spach MS Boineau JP. Microfibrosis produces electrical load variations due to loss of side-to-side cell connections: a major mechanism of structural heart disease arrhythmias. Pacing Clin Electrophysiol. (1997) 20:397–413. 10.1111/j.1540-8159.1997.tb06199.x 9058844

[37] de Bakker JM van Capelle FJ Janse MJ Tasseron S Vermeulen JT de Jonge N. Fractionated electrograms in dilated cardiomyopathy: origin and relation to abnormal conduction. J Am Coll Cardiol. (1996) 27:1071–8. 10.1016/0735-1097(95)00612-5 8609323

[38] Tomasek JJ Gabbiani G Hinz B Chaponnier C Brown RA. Myofibroblasts and mechano-regulation of connective tissue remodelling. Nat Rev Mol Cell Biol. (2002) 3:349–63. 10.1038/nrm809 11988769

[39] Sappino AP Schürch W Gabbiani G. Differentiation repertoire of fibroblastic cells: expression of cytoskeletal proteins as marker of phenotypic modulations. Lab Invest. (1990) 63:144–61. 2116562

[40] Willems IE Havenith MG De Mey JG Daemen MJ. The alpha-smooth muscle actin-positive cells in healing human myocardial scars. Am J Pathol. (1994) 145:868–75. 7943177 PMC1887334

[41] van Putten S Shafieyan Y Hinz B. Mechanical control of cardiac myofibroblasts. J Mol Cell Cardiol. (2016) 93:133–42. 10.1016/j.yjmcc.2015.11.025 26620422

[42] Travers JG Kamal FA Robbins J Yutzey KE Blaxall BC. Cardiac fibrosis: the fibroblast awakens. Circ Res. (2016) 118:1021–40. 10.1161/CIRCRESAHA.115.306565 26987915 PMC4800485

[43] Frangogiannis NG. Cardiac fibrosis: cell biological mechanisms, molecular pathways and therapeutic opportunities. Mol Aspects Med. (2019) 65:70–99. 10.1016/j.mam.2018.07.001 30056242

[44] Kurose H Mangmool S. Myofibroblasts and inflammatory cells as players of cardiac fibrosis. Arch Pharm Res. (2016) 39:1100–13. 10.1007/s12272-016-0809-6 27515051

[45] Baudino TA Carver W Giles W Borg TK. Cardiac fibroblasts: friend or foe? Am J Physiol Heart Circ Physiol. (2006) 291:H1015–26. 10.1152/ajpheart.00023.2006 16617141

[46] Fan D Takawale A Lee J Kassiri Z. Cardiac fibroblasts, fibrosis and extracellular matrix remodeling in heart disease. Fibrogene Tissue Repair. (2012) 5:15. 10.1186/1755-1536-5-15 22943504 PMC3464725

[47] Kuwahara F Kai H Tokuda K Kai M Takeshita A Egashira K. Transforming growth factor-β function blocking prevents myocardial fibrosis and diastolic dysfunction in pressure-overloaded rats. Circulation. (2002) 106:130–5. 10.1161/01.CIR.0000020689.12472.E0 12093782

[48] Ono K Ohtomo T Ninomiya-Tsuji J Tsuchiya M. A dominant negative TAK1 inhibits cellular fibrotic responses induced by TGF-β. Biochem Biophys Res Commun. (2003) 307:332–7. 10.1016/S0006-291X(03)01207-5 12859960

[49] See F Thomas W Way K Tzanidis A Kompa A Lewis D. p38 mitogen-activated protein kinase inhibition improves cardiac function and attenuates left ventricular remodeling following myocardial infarction in the rat. J Am Coll Cardiol. (2004) 44:1679–89. 10.1016/j.jacc.2004.07.038 15489104

[50] Tan SM Zhang Y Connelly KA Gilbert RE Kelly DJ. Targeted inhibition of activin receptor-like kinase 5 signaling attenuates cardiac dysfunction following myocardial infarction. Am J Physiol Heart Circ Physiol. (2010) 298:H1415–25. 10.1152/ajpheart.01048.2009 20154262

[51] Engebretsen KV Skårdal K Bjørnstad S Marstein HS Skrbic B Sjaastad I. Attenuated development of cardiac fibrosis in left ventricular pressure overload by SM16, an orally active inhibitor of ALK5. J Mol Cell Cardiol. (2014) 76:148–57. 10.1016/j.yjmcc.2014.08.008 25169971

[52] Lucas JA Zhang Y Li P Gong K Miller AP Hassan E. Inhibition of transforming growth factor-β signaling induces left ventricular dilation and dysfunction in the pressure-overloaded heart. Am J Physiol Heart Circ Physiol. (2010) 298:H424–32. 10.1152/ajpheart.00529.2009 19933419 PMC2822586

[53] Xavier S Piek E Fujii M Javelaud D Mauviel A Flanders KC. Amelioration of radiation-induced fibrosis: inhibition of transforming growth factor-β signaling by halofuginone. J Biol Chem. (2004) 279:15167–76. 10.1074/jbc.M309798200 14732719

[54] Shimada YJ Passeri JJ Baggish AL O'Callaghan C Lowry PA Yannekis G. Effects of losartan on left ventricular hypertrophy and fibrosis in patients with nonobstructive hypertrophic cardiomyopathy. JACC Heart Fail. (2013) 1:480–7. 10.1016/j.jchf.2013.09.001 24621999 PMC3950308

[55] Brilla CG Funck RC Rupp H. Lisinopril-mediated regression of myocardial fibrosis in patients with hypertensive heart disease. Circulation. (2000) 102:1388–93. 10.1161/01.CIR.102.12.1388 10993857

[56] Galie PA Russell MW Westfall MV Stegemann JP. Interstitial fluid flow and cyclic strain differentially regulate cardiac fibroblast activation via AT1R and TGF-β1. Exp Cell Res. (2012) 318:75–84. 10.1016/j.yexcr.2011.10.008 22020089 PMC3221916

[57] Singh AD Amit S Kumar OS Rajan M Mukesh N. Cardioprotective effects of bosentan, a mixed endothelin type A and B receptor antagonist, during myocardial ischaemia and reperfusion in rats. Basic Clin Pharmacol Toxicol. (2006) 98:604–10. 10.1111/j.1742-7843.2006.pto_405.x 16700825

[58] Ammarguellat F Larouche I Schiffrin EL. Myocardial fibrosis in DOCA-salt hypertensive rats: effect of endothelin ET_A_ receptor antagonism. Circulation. (2001) 103:319–24. 10.1161/01.CIR.103.2.319 11208696

[59] Wakeno M Minamino T Seguchi O Okazaki H Tsukamoto O Okada K. Long-term stimulation of adenosine A_2_B receptors begun after myocardial infarction prevents cardiac remodeling in rats. Circulation. (2006) 114:1923–32. 10.1161/CIRCULATIONAHA.106.630087 17043167

[60] Phosri S Arieyawong A Bunrukchai K Parichatikanond W Nishimura A Nishida M. Stimulation of adenosine A_2_B receptor inhibits endothelin-1-induced cardiac fibroblast proliferation and α-smooth muscle actin synthesis through the cAMP/Epac/PI3K/Akt-signaling pathway. Front Pharmacol. (2017) 8:428. 10.3389/fphar.2017.00428 28713274 PMC5492828

[61] Phosri S Bunrukchai K Parichatikanond W Sato VH Mangmool S. Epac is required for exogenous and endogenous stimulation of adenosine A_2_B receptor for inhibition of angiotensin II-induced collagen synthesis and myofibroblast differentiation. Purinergic Signal. (2018) 14:141–56. 10.1007/s11302-017-9600-5 29322373 PMC5940627

[62] Le Elizabeth D Pascotto M Leong-Poi H Sari I Micari A Kaul S. Anti-inflammatory and pro-angiogenic effects of beta blockers in a canine model of chronic ischemic cardiomyopathy: comparison between carvedilol and metoprolol. Basic Res Cardiol. (2013) 108:384. 10.1007/s00395-013-0384-7 24072434 PMC3867789

[63] Raake PW Vinge LE Gao E Boucher M Rengo G Chen X. G protein-coupled receptor kinase 2 ablation in cardiac myocytes before or after myocardial infarction prevents heart failure. Circ Res. (2008) 103:413–22. 10.1161/CIRCRESAHA.107.168336 18635825 PMC2679955

[64] Nuamnaichati N Sato VH Moongkarndi P Parichatikanond W Mangmool S. Sustained β-AR stimulation induces synthesis and secretion of growth factors in cardiac myocytes that affect on cardiac fibroblast activation. Life Sci. (2018) 193:257–69. 10.1016/j.lfs.2017.10.034 29107793

[65] Heldin CH Miyazono K ten Dijke P. TGF-β signalling from cell membrane to nucleus through SMAD proteins. Nature. (1997) 390:465–71. 10.1038/37284 9393997

[66] Roth DA Gold LI Han VK McCarthy JG Sung JJ Wisoff JH. Immunolocalization of transforming growth factor-β1, -β2, and -β3 and insulin-like growth factor I in premature cranial suture fusion. Plast Reconstr Surg. (1997) 99:300–9. 10.1097/00006534-199702000-00002 9030135

[67] Dobaczewski M Chen W Frangogiannis NG. Transforming growth factor (TGF)-β signaling in cardiac remodeling. J Mol Cell Cardiol. (2011) 51:600–6. 10.1016/j.yjmcc.2010.10.033 21059352 PMC3072437

[68] Shi M Zhu J Wang R Chen X Mi L Walz T. Latent TGF-β structure and activation. Nature. (2011) 474:343–9. 10.1038/nature10152 21677751 PMC4717672

[69] Wrana JL Attisano L Wieser R Ventura F Massagué J. Mechanism of activation of the TGF-β receptor. Nature. (1994) 370:341–7. 10.1038/370341a0 8047140

[70] Loomans HA Andl CD. Activin receptor-like kinases: a diverse family playing an important role in cancer. Am J Cancer Res. (2016) 6:2431–47. 27904762 PMC5126264

[71] Euler-Taimor G Heger J. The complex pattern of SMAD signaling in the cardiovascular system. Cardiovasc Res. (2006) 69:15–25. 10.1016/j.cardiores.2005.07.007 16107248

[72] Khalil H Kanisicak O Prasad V Correll RN Fu X Schips T. Fibroblast-specific TGF-β-Smad2/3 signaling underlies cardiac fibrosis. J Clin Invest. (2017) 127:3770–83. 10.1172/JCI94753 28891814 PMC5617658

[73] Zhao M Mishra L Deng CX. The role of TGF-β/SMAD4 signaling in cancer. Int J Biol Sci. (2018) 14:111–23. 10.7150/ijbs.23230 29483830 PMC5821033

[74] Yan X Liao H Cheng M Shi X Lin X Feng XH. Smad7 protein interacts with receptor-regulated Smads (R-Smads) to inhibit transforming growth factor-β (TGF-β)/Smad signaling. J Biol Chem. (2016) 291:382–92. 10.1074/jbc.M115.694281 26555259 PMC4697173

[75] Zhang YE. Non-Smad signaling pathways of the TGF-β family. Cold Spring Harb Perspect Biol. (2017) 9:a022129. 10.1101/cshperspect.a022129 27864313 PMC5287080

[76] Xie L Law BK Chytil AM Brown KA Aakre ME Moses HL. Activation of the Erk pathway is required for TGF-β1-induced EMT *in vitro*. Neoplasia. (2004) 6:603–10. 10.1593/neo.04241 15548370 PMC1531665

[77] Bakin AV Rinehart C Tomlinson AK Arteaga CL. p38 mitogen-activated protein kinase is required for TGFβ-mediated fibroblastic transdifferentiation and cell migration. J Cell Sci. (2002) 115:3193–206. Retrived from: https://jcs.biologists.org/content/115/15/3193 12118074 10.1242/jcs.115.15.3193

[78] Yu L Hébert MC Zhang YE. TGF-β receptor-activated p38 MAP kinase mediates Smad-independent TGF-β responses. EMBO J. (2002) 21:3749–59. 10.1093/emboj/cdf366 12110587 PMC126112

[79] Ozdamar B Bose R Barrios-Rodiles M Wang HR Zhang Y Wrana JL. Regulation of the polarity protein Par6 by TGFβ receptors controls epithelial cell plasticity. Science. (2005) 307:1603–9. 10.1126/science.1105718 15761148

[80] Bakin AV Tomlinson AK Bhowmick NA Moses HL Arteaga CL. Phosphatidylinositol 3-kinase function is required for transforming growth factor-β-mediated epithelial to mesenchymal transition and cell migration. J Biol Chem. (2000) 275:36803–10. 10.1074/jbc.M005912200 10969078

[81] Lamouille S Derynck R. Cell size and invasion in TGF-β-induced epithelial to mesenchymal transition is regulated by activation of the mTOR pathway. J Cell Biol. (2007) 178:437–51. 10.1083/jcb.200611146 17646396 PMC2064840

[82] Suzuki HI. MicroRNA control of TGF-β signaling. Int J Mol Sci. (2018) 19:1901. 10.3390/ijms19071901 29958433 PMC6073626

[83] Robson CN Gnanapragasam V Byrne RL Collins AT Neal DE. Transforming growth factor-β1 up-regulates p15, p21 and p27 and blocks cell cycling in G1 in human prostate epithelium. J Endocrinol. (1999) 160:257–66. 10.1677/joe.0.1600257 9924195

[84] Yagi K Furuhashi M Aoki H Goto D Kuwano H Sugamura K. C-myc is a downstream target of the smad pathway. J Biol Chem. (2002) 277:854–61. 10.1074/jbc.M104170200 11689553

[85] Spender LC O'Brien DI Simpson D Dutt D Gregory CD Allday MJ. TGF-β induces apoptosis in human B cells by transcriptional regulation of BIK and BCL-XL. Cell Death Differ. (2009) 16:593–602. 10.1038/cdd.2008.183 19136942 PMC2857326

[86] Kim SG Jong HS Kim TY Lee JW Kim NK Hong SH. Transforming growth factor-β1 induces apoptosis through Fas ligand-independent activation of the Fas death pathway in human gastric SNU-620 carcinoma cells. Mol Biol Cell. (2004) 15:420–34. 10.1091/mbc.e03-04-0201 14595120 PMC329198

[87] Zonneville J Safina A Truskinovsky AM Arteaga CL Bakin AV. TGF-β signaling promotes tumor vasculature by enhancing the pericyte-endothelium association. BMC Cancer. (2018) 18:670. 10.1186/s12885-018-4587-z 29921235 PMC6008941

[88] Smith AL Iwanaga R Drasin DJ Micalizzi DS Vartuli RL Tan AC. The miR-106b-25 cluster targets Smad7, activates TGF-β signaling, and induces EMT and tumor initiating cell characteristics downstream of six1 in human breast cancer. Oncogene. (2012) 31:5162–71. 10.1038/onc.2012.11 22286770 PMC3342483

[89] Metelli A Wu BX Fugle CW Rachidi S Sun S Zhang Y. Surface expression of TGFβ docking receptor GARP promotes oncogenesis and immune tolerance in breast cancer. Cancer Res. (2016) 76:7106–17. 10.1158/0008-5472.CAN-16-1456 27913437 PMC5504525

[90] Takasaka N Seed RI Cormier A Bondesson AJ Lou J Elattma A. Integrin αvβ8-expressing tumor cells evade host immunity by regulating TGF-β activation in immune cells. JCI Insight. (2018) 3:e122591. 10.1172/jci.insight.122591 30333313 PMC6237456

[91] Fattovich G Stroffolini T Zagni I Donato F. Hepatocellular carcinoma in cirrhosis: incidence and risk factors. Gastroenterology. (2004) 127:S35–50. 10.1053/j.gastro.2004.09.014 15508101

[92] Taguchi S. Comprehensive review of the epidemiology and treatments for malignant adult cardiac tumors. Gen Thorac Cardiovasc Surg. (2018) 66:257–62. 10.1007/s11748-018-0912-3 29594875

[93] Frangogiannis NG. The role of transforming growth factor (TGF)-β in the infarcted myocardium. J Thorac Dis. (2017) 9(Suppl. 1):S52–63. 10.21037/jtd.2016.11.19 28446968 PMC5383562

[94] Dewald O Ren G Duerr GD Zoerlein M Klemm C Gersch C. Of mice and dogs: species-specific differences in the inflammatory response following myocardial infarction. Am J Pathol. (2004) 164:665–77. 10.1016/S0002-9440(10)63154-9 14742270 PMC1602262

[95] van Amerongen MJ Harmsen MC van Rooijen N Petersen AH van Luyn MJ. Macrophage depletion impairs wound healing and increases left ventricular remodeling after myocardial injury in mice. Am J Pathol. (2007) 170:818–29. 10.2353/ajpath.2007.060547 17322368 PMC1864893

[96] Wünsch M Sharma HS Markert T Bernotat-Danielowski S Schott RJ Kremer P. *In situ* localization of transforming growth factor-β1 in porcine heart: enhanced expression after chronic coronary artery constriction. J Mol Cell Cardiol. (1991) 23:1051–62. 10.1016/0022-2828(91)91640-D 1942095

[97] Birdsall HH Green DM Trial J Youker KA Burns AR MacKay CR. Complement C5a, TGF-β1, and MCP-1, in sequence, induce migration of monocytes into ischemic canine myocardium within the first one to five hours after reperfusion. Circulation. (1997) 95:684–92. 10.1161/01.CIR.95.3.684 9024158

[98] Sarrazy V Koehler A Chow ML Zimina E Li CX Kato H. Integrins αvβ5 and αvβ3 promote latent TGF-β1 activation by human cardiac fibroblast contraction. Cardiovasc Res. (2014) 102:407–17. 10.1093/cvr/cvu053 24639195 PMC4030512

[99] Frangogiannis NG Ren G Dewald O Zymek P Haudek S Koerting A. Critical role of endogenous thrombospondin-1 in preventing expansion of healing myocardial infarcts. Circulation. (2005) 111:2935–42. 10.1161/CIRCULATIONAHA.104.510354 15927970

[100] Lefer AM Tsao P Aoki N Palladino MA Jr. Mediation of cardioprotection by transforming growth factor-β. Science. (1990) 249:61–4. 10.1126/science.2164258 2164258

[101] Baxter GF Mocanu MM Brar BK Latchman DS Yellon DM. Cardioprotective effects of transforming growth factor-β1 during early reoxygenation or reperfusion are mediated by p42/p44 MAPK. J Cardiovasc Pharmacol. (2001) 38:930–9. 10.1097/00005344-200112000-00015 11707697

[102] Schröder D Heger J Piper HM Euler G. Angiotensin II stimulates apoptosis via TGF-β1 signaling in ventricular cardiomyocytes of rat. J Mol Med. (2006) 84:975–83. 10.1007/s00109-006-0090-0 16924465

[103] Celada A Maki RA. Transforming growth factor-β enhances the M-CSF and GM-CSF-stimulated proliferation of macrophages. J Immunol. (1992) 148:1102–5. 1737928

[104] Gorelik L Flavell RA. Abrogation of TGFβ signaling in T cells leads to spontaneous T cell differentiation and autoimmune disease. Immunity. (2000) 12:171–81. 10.1016/S1074-7613(00)80170-3 10714683

[105] Dobaczewski M Bujak M Li N Gonzalez-Quesada C Mendoza LH Wang XF. Smad3 signaling critically regulates fibroblast phenotype and function in healing myocardial infarction. Circ Res. (2010) 107:418–28. 10.1161/CIRCRESAHA.109.216101 20522804 PMC2917472

[106] Bujak M Ren G Kweon HJ Dobaczewski M Reddy A Taffet G. Essential role of Smad3 in infarct healing and in the pathogenesis of cardiac remodeling. Circulation. (2007) 116:2127–38. 10.1161/CIRCULATIONAHA.107.704197 17967775

[107] Krenning G Zeisberg EM Kalluri R. The origin of fibroblasts and mechanism of cardiac fibrosis. J Cell Physiol. (2010) 225:631–7. 10.1002/jcp.22322 20635395 PMC3098503

[108] Duncan MR Frazier KS Abramson S Williams S Klapper H Huang X. Connective tissue growth factor mediates transforming growth factor-β-induced collagen synthesis: down-regulation by cAMP. FASEB J. (1999) 13:1774–86. 10.1096/fasebj.13.13.1774 10506580

[109] Eghbali M Tomek R Sukhatme VP Woods C Bhambi B. Differential effects of transforming growth factor-β1 and phorbol myristate acetate on cardiac fibroblasts. regulation of fibrillar collagen mRNAs and expression of early transcription factors. Circ Res. (1991) 69:483–90. 10.1161/01.RES.69.2.483 1860186

[110] Matsumoto-Ida M Takimoto Y Aoyama T Akao M Takeda T Kita T. Activation of TGF-β1-TAK1-p38 MAPK pathway in spared cardiomyocytes is involved in left ventricular remodeling after myocardial infarction in rats. Am J Physiol Heart Circ Physiol. (2006) 290:H709–15. 10.1152/ajpheart.00186.2005 16183734

[111] Wang S Wilkes MC Leof EB Hirschberg R. Imatinib mesylate blocks a non-Smad TGF-β pathway and reduces renal fibrogenesis *in vivo*. FASEB J. (2005) 19:1–11. 10.1096/fj.04-2370com 15629889

[112] Daniels CE Wilkes MC Edens M Kottom TJ Murphy SJ Limper AH. Imatinib mesylate inhibits the profibrogenic activity of TGF-β and prevents bleomycin-mediated lung fibrosis. J Clin Invest. (2004) 114:1308–16. 10.1172/JCI200419603 15520863 PMC524221

[113] Seeland U Haeuseler C Hinrichs R Rosenkranz S Pfitzner T Scharffetter-Kochanek K. Myocardial fibrosis in transforming growth factor-β1 (TGF-β1) transgenic mice is associated with inhibition of interstitial collagenase. Eur J Clin Invest. (2002) 32:295–303. 10.1046/j.1365-2362.2002.00985.x 12027867

[114] Frangogiannis NG. The immune system and the remodeling infarcted heart: cell biological insights and therapeutic opportunities. J Cardiovasc Pharmacol. (2014) 63:185–95. 10.1097/FJC.0000000000000003 24072174 PMC3949163

[115] Zeisberg EM Tarnavski O Zeisberg M Dorfman AL McMullen JR Gustafsson E. Endothelial-to-mesenchymal transition contributes to cardiac fibrosis. Nat Med. (2007) 13:952–61. 10.1038/nm1613 17660828

[116] Chen Y Di C Zhang X Wang J Wang F Yan JF Transforming growth factor β signaling pathway: a promising therapeutic target for cancer. J Cell Physiol. (2020) 253:1903–14. 10.1002/jcp.29108 31332789

[117] Anderton MJ Mellor HR Bell A Sadler C Pass M Powell S. Induction of heart valve lesions by small-molecule ALK5 inhibitors. Toxicol Pathol. (2011) 39:916–24. 10.1177/0192623311416259 21859884

[118] Neuzillet C Tijeras-Raballand A Cohen R Cros J Faivre S Raymond E. Targeting the TGFβ pathway for cancer therapy. Pharmacol Ther. (2015) 147:22–31. 10.1016/j.pharmthera.2014.11.001 25444759

[119] Hau P Jachimczak P Schlingensiepen R Schulmeyer F Jauch T Steinbrecher A. Inhibition of TGF-β2 with AP 12009 in recurrent malignant gliomas: from preclinical to phase I/II studies. Oligonucleotides. (2007) 17:201–12. 10.1089/oli.2006.0053 17638524

[120] Zhang C Zhang F Tsan R Fidler IJ. Transforming growth factor-β2 is a molecular determinant for site-specific melanoma metastasis in the brain. Cancer Res. (2009) 69:828–35. 10.1158/0008-5472.CAN-08-2588 19141644 PMC2633423

[121] Jonson T Albrechtsson E Axelson J Heidenblad M Gorunova L Johansson B. Altered expression of TGFβ receptors and mitogenic effects of TGFβ in pancreatic carcinomas. Int J Oncol. (2001) 19:71–81. 10.3892/ijo.19.1.71 11408925

[122] Schlingensiepen KH Jaschinski F Lang SA Moser C Geissler EK Schlitt HJ. Transforming growth factor-β2 gene silencing with trabedersen (AP 12009) in pancreatic cancer. Cancer Sci. (2011) 102:1193–200. 10.1111/j.1349-7006.2011.01917.x 21366804

[123] Bellone G Carbone A Tibaudi D Mauri F Ferrero I Smirne C. Differential expression of transforming growth factors-β1, -β2 and -β3 in human colon carcinoma. Eur J Cancer. (2001) 37:224–33. 10.1016/S0959-8049(00)00391-9 11166150

[124] Rowland-Goldsmith MA Maruyama H Kusama T Ralli S Korc M. Soluble type II transforming growth factor-beta (TGF-β) receptor inhibits TGF-β signaling in COLO-357 pancreatic cancer cells *in vitro* and attenuates tumor formation. Clin Cancer Res. (2001) 7:2931–40. Retrived from: https://clincancerres.aacrjournals.org/content/7/9/2931.article-info 11555612

[125] Sakaguchi J Kyo S Kanaya T Maida Y Hashimoto M Nakamura M. Aberrant expression and mutations of TGF-β receptor type II gene in endometrial cancer. Gynecol Oncol. (2005) 98:427–33. 10.1016/j.ygyno.2005.04.031 15993480

[126] Copland JA Luxon BA Ajani L Maity T Campagnaro E Guo H. Genomic profiling identifies alterations in TGFβ signaling through loss of TGFβ receptor expression in human renal cell carcinogenesis and progression. Oncogene. (2003) 22:8053–62. 10.1038/sj.onc.1206835 12970754

[127] Finger EC Turley RS Dong M How T Fields TA Blobe GC. TβRIII suppresses non-small cell lung cancer invasiveness and tumorigenicity. Carcinogenesis. (2008) 29:528–35. 10.1093/carcin/bgm289 18174241

[128] Bandyopadhyay A López-Casillas F Malik SN Montiel JL Mendoza V Yang J. Antitumor activity of a recombinant soluble betaglycan in human breast cancer xenograft. Cancer Res. (2002) 62:4690–5. Retrived from: https://cancerres.aacrjournals.org/content/62/16/4690 12183427

[129] Calon A Lonardo E Berenguer-Llergo A Espinet E Hernando-Momblona X Iglesias M. Stromal gene expression defines poor-prognosis subtypes in colorectal cancer. Nat Genet. (2015) 47:320–9. 10.1038/ng.3225 25706628

[130] Alsina-Sanchis E Figueras A Lahiguera Á Vidal A Casanovas O Graupera M. The TGFβ pathway stimulates ovarian cancer cell proliferation by increasing IGF1R levels. Int J Cancer. (2016) 139:1894–903. 10.1002/ijc.30233 27299695

[131] Dituri F Mazzocca A Fernando J Peidrò FJ Papappicco P Fabregat I. Differential inhibition of the TGF-β signaling pathway in HCC cells using the small molecule inhibitor LY2157299 and the D10 monoclonal antibody against TGF-β receptor type II. PLoS ONE. (2013) 8:e67109. 10.1371/journal.pone.0067109 23826206 PMC3694933

[132] Dzieran J Fabian J Feng T Coulouarn C Ilkavets I Kyselova A. Comparative analysis of TGF-β/Smad signaling dependent cytostasis in human hepatocellular carcinoma cell lines. PLoS ONE. (2013) 8:e72252. 10.1371/journal.pone.0072252 23991075 PMC3750029

[133] Serova M Tijeras-Raballand A Dos Santos C Albuquerque M Paradis V Neuzillet C. Effects of TGF-β signalling inhibition with galunisertib (LY2157299) in hepatocellular carcinoma models and in *ex vivo* whole tumor tissue samples from patients. Oncotarget. (2015) 6:21614–27. 10.18632/oncotarget.4308 26057634 PMC4673290

[134] Son JY Park SY Kim SJ Lee SJ Park SA Kim MJ. EW-7197, a novel ALK-5 kinase inhibitor, potently inhibits breast to lung metastasis. Mol Cancer Ther. (2014) 13:1704–16. 10.1158/1535-7163.MCT-13-0903 24817629

[135] Park CY Son JY Jin CH Nam JS Kim DK Sheen YY. EW-7195, a novel inhibitor of ALK5 kinase inhibits EMT and breast cancer metastasis to lung. Eur J Cancer. (2011) 47:2642–53. 10.1016/j.ejca.2011.07.007 21852112

[136] Zhang B Halder SK Zhang S Datta PK. Targeting transforming growth factor-β signaling in liver metastasis of colon cancer. Cancer Lett. (2009) 277:114–20. 10.1016/j.canlet.2008.11.035 19147275 PMC2776056

[137] Mazzocca A Fransvea E Lavezzari G Antonaci S Giannelli G. Inhibition of transforming growth factor-β receptor I kinase blocks hepatocellular carcinoma growth through neo-angiogenesis regulation. Hepatology. (2009) 50:1140–51. 10.1002/hep.23118 19711426

[138] Ge R Rajeev V Ray P Lattime E Rittling S Medicherla S. Inhibition of growth and metastasis of mouse mammary carcinoma by selective inhibitor of transforming growth factor-β type I receptor kinase *in vivo*. Clin Cancer Res. (2006) 12:4315–30. 10.1158/1078-0432.CCR-06-0162 16857807

[139] Gaspar NJ Li L Kapoun AM Medicherla S Reddy M Li G. Inhibition of transforming growth factor-β signaling reduces pancreatic adenocarcinoma growth and invasiveness. Mol Pharmacol. (2007) 72:152–61. 10.1124/mol.106.029025 17400764

[140] Bogdahn U Hau P Stockhammer G Venkataramana NK Mahapatra AK Suri A. Targeted therapy for high-grade glioma with the TGF-β2 inhibitor trabedersen: results of a randomized and controlled phase IIb study. Neuro Oncol. (2011) 13:132–42. 10.1093/neuonc/noq142 20980335 PMC3018908

[141] Nemunaitis J Dillman RO Schwarzenberger PO Senzer. Phase II study of belagenpumatucel-L, a transforming growth factor beta-2 antisense gene-modified allogeneic tumor cell vaccine in non-small-cell lung cancer. J Clin Oncol. (2006) 24:4721–30. 10.1200/JCO.2005.05.5335 16966690

[142] Nemunaitis J Nemunaitis M Senzer N Snitz P Bedell C Kumar P. Phase II trial of Belagenpumatucel-L, a TGF-β2 antisense gene modified allogeneic tumor vaccine in advanced non small cell lung cancer (NSCLC) patients. Cancer Gene Ther. (2009) 16:620–4. 10.1038/cgt.2009.15 19287371

[143] Giaccone G Bazhenova LA Nemunaitis J Tan M Juhász E Ramlau R. A phase III study of belagenpumatucel-L, an allogeneic tumour cell vaccine, as maintenance therapy for non-small cell lung cancer. Eur J Cancer. (2015) 51:2321–9. 10.1016/j.ejca.2015.07.035 26283035

[144] Stevenson JP Kindler HL Papasavvas E Sun J Jacobs-Small M Hull J. Immunological effects of the TGFβ-blocking antibody GC1008 in malignant pleural mesothelioma patients. Oncoimmunology. (2013) 2:e26218. 10.4161/onci.26218 24179709 PMC3812201

[145] Formenti SC Lee P Adams S Goldberg JD Li X Xie MW. Focal irradiation and systemic TGFβ blockade in metastatic breast cancer. Clin Cancer Res. (2018) 24:2493–504. 10.1158/1078-0432.CCR-17-3322 29476019 PMC5999326

[146] Melisi D Garcia-Carbonero R Macarulla T Pezet D Deplanque G Fuchs M. Galunisertib plus gemcitabine vs. gemcitabine for first-line treatment of patients with unresectable pancreatic cancer. Br J Cancer. (2018) 119:1208–14. 10.1038/s41416-018-0246-z 30318515 PMC6251034

[147] Faivre SJ Santoro A Kelley RK Merle P Gane E Douillard JY A phase 2 study of a novel transforming growth factor-β (TGF-β) receptor I kinase inhibitor, LY2157299 monohydrate (LY), in patients with advanced hepatocellular carcinoma (HCC). J Clin Oncol. (2014) 32:173 10.1200/jco.2014.32.3_suppl.lba173

[148] Ryan CW Matias C Agulnik M Lopez-Pousa A Williams C de Alwis DP. A phase II study of tasisulam sodium (LY573636 sodium) as second-line or third-line treatment for patients with unresectable or metastatic soft tissue sarcoma. Invest New Drugs. (2013) 31:145–51. 10.1007/s10637-012-9819-5 22539091

[149] Schlingensiepen KH Schlingensiepen R Steinbrecher A Hau P Bogdahn U Fischer-Blass B. Targeted tumor therapy with the TGF-β2 antisense compound AP 12009. Cytokine Growth Factor Rev. (2006) 17:129–39. 10.1016/j.cytogfr.2005.09.002 16377233

[150] David JM Dominguez C Palena C. Pharmacological and immunological targeting of tumor mesenchymalization. Pharmacol Ther. (2017) 170:212–25. 10.1016/j.pharmthera.2016.11.011 27916651 PMC5274580

[151] Morris JC Tan AR Olencki TE Shapiro GI Dezube BJ Reiss M. Phase I study of GC1008 (fresolimumab): a human anti-transforming growth factor-β (TGFβ) monoclonal antibody in patients with advanced malignant melanoma or renal cell carcinoma. PLoS ONE. (2014) 9:e90353. 10.1371/journal.pone.0090353 24618589 PMC3949712

[152] Holmgaard RB Schaer DA Li Y Castaneda SP Murphy MY Xu X. Targeting the TGFβ pathway with galunisertib, a TGFβRI small molecule inhibitor, promotes anti-tumor immunity leading to durable, complete responses, as monotherapy and in combination with checkpoint blockade. J Immunother Cancer. (2018) 6:47. 10.1186/s40425-018-0356-4 29866156 PMC5987416

[153] Mariathasan S Turley SJ Nickles D Castiglioni A Yuen K Wang Y. TGFβ attenuates tumour response to PD-L1 blockade by contributing to exclusion of T cells. Nature. (2018) 554:544–8. 10.1038/nature25501 29443960 PMC6028240

[154] de Gramont A Faivre S Raymond E. Novel TGF-β inhibitors ready for prime time in onco-immunology. Oncoimmunology. (2017) 6:e1257453. 10.1080/2162402X.2016.1257453 28197376 PMC5283641

[155] Rodon J Carducci MA Sepulveda-Sánchez JM Azaro A Calvo E Seoane J. First-in-human dose study of the novel transforming growth factor-β receptor I kinase inhibitor LY2157299 monohydrate in patients with advanced cancer and glioma. Clin Cancer Res. (2015) 21:553–60. 10.1158/1078-0432.CCR-14-1380 25424852 PMC4337847

[156] Kovacs RJ Maldonado G Azaro A Fernández MS Romero FL Sepulveda-Sánchez JM. Cardiac safety of TGF-β receptor I kinase inhibitor LY2157299 monohydrate in cancer patients in a first-in-human dose study. Cardiovasc Toxicol. (2015) 15:309–23. 10.1007/s12012-014-9297-4 25488804 PMC4575352

[157] Gueorguieva I Cleverly AL Stauber A Sada Pillay N Rodon JA Miles CP. Defining a therapeutic window for the novel TGF-β inhibitor LY2157299 monohydrate based on a pharmacokinetic/pharmacodynamic model. Br J Clin Pharmacol. (2014) 77:796–807. 10.1111/bcp.12256 24868575 PMC4004400

[158] Park SA Kim MJ Park SY Kim JS Lee SJ Woo HA. EW-7197 inhibits hepatic, renal, and pulmonary fibrosis by blocking TGF-β/Smad and ROS signaling. Cell Mol Life Sci. (2015) 72:2023–39. 10.1007/s00018-014-1798-6 25487606 PMC11113926

[159] Song KM Chung DY Choi MJ Ghatak K Nguyen NM Limanjaya A. Vactosertib, a novel, orally bioavailable activin receptor-like kinase 5 inhibitor, promotes regression of fibrotic plaques in a rat model of Peyronie's disease. World J Mens Health. (2019). 10.5534/wjmh.190071. [Epub ahead of print]. 31496148 PMC7502315

[160] Lan Y Zhang D Xu C Hance KW Marelli B Qi J. Enhanced preclinical antitumor activity of M7824, a bifunctional fusion protein simultaneously targeting PD-L1 and TGF-β. Sci Transl Med. (2018) 10:eaan5488. 10.1126/scitranslmed.aan5488 29343622

[161] Fang L Murphy AJ Dart AM. A clinical perspective of anti-fibrotic therapies for cardiovascular disease. Front Pharmacol. (2017) 8:186. 10.3389/fphar.2017.00186 28428753 PMC5382201

[162] Tomita H Egashira K Ohara Y Takemoto M Koyanagi M Katoh M. Early induction of transforming growth factor-β via angiotensin II type 1 receptors contributes to cardiac fibrosis induced by long-term blockade of nitric oxide synthesis in rats. Hypertension. (1998) 32:273–9. 10.1161/01.HYP.32.2.273 9719054

[163] Frantz S Hu K Adamek A Wolf J Sallam A Maier SK. Transforming growth factor-β inhibition increases mortality and left ventricular dilatation after myocardial infarction. Basic Res Cardiol. (2008) 103:485–92. 10.1007/s00395-008-0739-7 18651091

[164] Ihn H Yamane K Kubo M Tamaki K. Blockade of endogenous transforming growth factor-β signaling prevents up-regulated collagen synthesis in scleroderma fibroblasts: association with increased expression of transforming growth factor-β receptors. Arthritis Rheum. (2001) 44:474–80. 10.1002/1529-0131(200102)44:2<474::AID-ANR67>3.0.CO;2-# 11229480

[165] Okada H Takemura G Kosai K Li Y Takahashi T Esaki M. Postinfarction gene therapy against transforming growth factor-β signal modulates infarct tissue dynamics and attenuates left ventricular remodeling and heart failure. Circulation. (2005) 111:2430–7. 10.1161/01.CIR.0000165066.71481.8E 15867170

[166] Liao P Georgakopoulos D Kovacs A Zheng M Lerner D Pu H. The *in vivo* role of p38 MAP kinases in cardiac remodeling and restrictive cardiomyopathy. Proc Natl Acad Sci USA. (2001) 98:12283–8. 10.1073/pnas.211086598 11593045 PMC59806

[167] Yan W Wang P Zhao CX Tang J Xiao X Wang DW. Decorin gene delivery inhibits cardiac fibrosis in spontaneously hypertensive rats by modulation of transforming growth factor-β/Smad and p38 mitogen- activated protein kinase signaling pathways. Hum Gene Ther. (2009) 20:1190–200. 10.1089/hum.2008.204 19697998

[168] Zhang D Gaussin V Taffet GE Belaguli NS Yamada M Schwartz RJ. TAK1 is activated in the myocardium after pressure overload and is sufficient to provoke heart failure in transgenic mice. Nat Med. (2000) 6:556–63. 10.1038/75037 10802712

[169] Park S Nguyen NB Pezhouman A Ardehali R. Cardiac fibrosis: potential therapeutic targets. Transl Res. (2019) 209:121–37. 10.1016/j.trsl.2019.03.001 30930180 PMC6545256

[170] de Oliveira FL Araújo-Jorge TC de Souza EM de Oliveira GM Degrave WM Feige JJ. Oral administration of GW788388, an inhibitor of transforming growth factor-β signaling, prevents heart fibrosis in Chagas disease. PLoS Negl Trop Dis. (2012) 6:e1696. 10.1371/journal.pntd.0001696 22720109 PMC3373641

[171] Derangeon M Montnach J Cerpa CO Jagu B Patin J Toumaniantz G. Transforming growth factor-β receptor inhibition prevents ventricular fibrosis in a mouse model of progressive cardiac conduction disease. Cardiovasc Res. (2017) 113:464–74. 10.1093/cvr/cvx026 28339646

[172] Mirkovic S Seymour AM Fenning A Strachan A Margolin SB Taylor SM. Attenuation of cardiac fibrosis by pirfenidone and amiloride in DOCA-salt hypertensive rats. Br J Pharmacol. (2002) 135:961–8. 10.1038/sj.bjp.0704539 11861324 PMC1573203

[173] Nguyen DT Ding C Wilson E Marcus GM Olgin JE. Pirfenidone mitigates left ventricular fibrosis and dysfunction after myocardial infarction and reduces arrhythmias. Heart Rhythm. (2010) 7:1438–45. 10.1016/j.hrthm.2010.04.030 20433946

[174] Wang Y Wu Y Chen J Zhao S Li H. Pirfenidone attenuates cardiac fibrosis in a mouse model of TAC-induced left ventricular remodeling by suppressing NLRP3 inflammasome formation. Cardiology. (2013) 126:1–11. 10.1159/000351179 23839341

[175] Yamagami K Oka T Wang Q Ishizu T Lee JK Miwa K. Pirfenidone exhibits cardioprotective effects by regulating myocardial fibrosis and vascular permeability in pressure-overloaded hearts. Am J Physiol Heart Circ Physiol. (2015) 309:H512–22. 10.1152/ajpheart.00137.2015 26055790

[176] Miric G Dallemagne C Endre Z Margolin S Taylor SM Brown L. Reversal of cardiac and renal fibrosis by pirfenidone and spironolactone in streptozotocin-diabetic rats. Br J Pharmacol. (2001) 133:687–94. 10.1038/sj.bjp.0704131 11429393 PMC1572838

[177] Martin J Kelly DJ Mifsud SA Zhang Y Cox AJ See F. Tranilast attenuates cardiac matrix deposition in experimental diabetes: role of transforming growth factor-β. Cardiovasc Res. (2005) 65:694–701. 10.1016/j.cardiores.2004.10.041 15664396

[178] Kelly DJ Zhang Y Connelly K Cox AJ Martin J Krum H. Tranilast attenuates diastolic dysfunction and structural injury in experimental diabetic cardiomyopathy. Am J Physiol Heart Circ Physiol. (2007) 293:H2860–9. 10.1152/ajpheart.01167.2006 17720766

[179] Kagitani S Ueno H Hirade S Takahashi T Takata M Inoue H. Tranilast attenuates myocardial fibrosis in association with suppression of monocyte/macrophage infiltration in DOCA/salt hypertensive rats. J Hypertens. (2004) 22:1007–15. 10.1097/00004872-200405000-00024 15097242

[180] Hocher B Godes M Olivier J Weil J Eschenhagen T Slowinski T. Inhibition of left ventricular fibrosis by tranilast in rats with renovascular hypertension. J Hypertens. (2002) 20:745–51. 10.1097/00004872-200204000-00034 11910312

[181] See F Watanabe M Kompa AR Wang BH Boyle AJ Kelly DJ. Early and delayed tranilast treatment reduces pathological fibrosis following myocardial infarction. Heart Lung Circ. (2013) 22:122–32. 10.1016/j.hlc.2012.08.054 22986349

[182] Pinto YM Pinto-Sietsma SJ Philipp T Engler S Kossamehl P Hocher B. Reduction in left ventricular messenger RNA for transforming growth factor-β1 attenuates left ventricular fibrosis and improves survival without lowering blood pressure in the hypertensive TGR(mRen2)27 rat. Hypertension. (2000) 36:747–54. 10.1161/01.HYP.36.5.747 11082138

[183] Holmes DJ Savage M LaBlanche JM Grip L Serruys PW Fitzgerald P. Results of prevention of REStenosis with tranilast and its outcomes (PRESTO) trial. Circulation. (2002) 106:1243–50. 10.1161/01.CIR.0000028335.31300.DA 12208800

[184] Gellibert F de Gouville AC Woolven J Mathews N Nguyen VL Bertho-Ruault C. Discovery of 4-{4-[3-(pyridin-2-yl)-1H-pyrazol-4-yl]pyridin-2-yl}-N-(tetrahydro-2H- pyran-4-yl)benzamide (GW788388): a potent, selective, and orally active transforming growth factor-β type I receptor inhibitor. J Med Chem. (2006) 49:2210–21. 10.1021/jm0509905 16570917

[185] Petersen M Thorikay M Deckers M van Dinther M Grygielko ET Gellibert F. Oral administration of GW788388, an inhibitor of TGF-β type I and II receptor kinases, decreases renal fibrosis. Kidney Int. (2008) 73:705–15. 10.1038/sj.ki.5002717 18075500

[186] King TE Bradford WZ Castro-Bernardini S Fagan EA Glaspole I Glassberg MK. A phase 3 trial of pirfenidone in patients with idiopathic pulmonary fibrosis. N Engl J Med. (2014) 370:2083–92. 10.1056/NEJMoa1402582 24836312

[187] Iyer SN Gurujeyalakshmi G Giri SN. Effects of pirfenidone on transforming growth factor-β gene expression at the transcriptional level in bleomycin hamster model of lung fibrosis. J Pharmacol Exp Ther. (1999) 291:367–73. 10490926

[188] Lewis GA Schelbert EB Naish JH Bedson E Dodd S Eccleson H. Pirfenidone in heart failure with preserved ejection fraction-rationale and design of the PIROUETTE trial. Cardiovasc Drugs Ther. (2019) 33:461–70. 10.1007/s10557-019-06876-y 31069575 PMC6689029

[189] Tamai H Katoh O Suzuki S Fujii K Aizawa T Takase S. Impact of tranilast on restenosis after coronary angioplasty: tranilast restenosis following angioplasty trial (TREAT). Am Heart J. (1999) 138:968–75. 10.1016/S0002-8703(99)70025-6 10539831

